# Autistic Adult Health and Professional Perceptions of It: Evidence From the ASDEU Project

**DOI:** 10.3389/fpsyt.2021.614102

**Published:** 2021-05-28

**Authors:** Martina Micai, Antonio Ciaramella, Tommaso Salvitti, Francesca Fulceri, Laura Maria Fatta, Luise Poustka, Robert Diehm, Georgi Iskrov, Rumen Stefanov, Quentin Guillon, Bernadette Rogé, Anthony Staines, Mary Rose Sweeney, Andrew Martin Boilson, Thora Leósdóttir, Evald Saemundsen, Irma Moilanen, Hanna Ebeling, Anneli Yliherva, Mika Gissler, Tarja Parviainen, Pekka Tani, Rafal Kawa, Astrid Vicente, Célia Rasga, Magdalena Budişteanu, Ian Dale, Carol Povey, Noelia Flores, Cristina Jenaro, Maria Luisa Monroy, Patricia García Primo, Tony Charman, Susanne Cramer, Christine Kloster Warberg, Ricardo Canal-Bedia, Manuel Posada, Maria Luisa Scattoni, Diana Schendel

**Affiliations:** ^1^Istituto Superiore di Sanità, Research Coordination and Support Service, Rome, Italy; ^2^Department of Child and Adolescent Psychiatry and Psychotherapy, University Medical Center Göttingen, Gottingen, Germany; ^3^Department of Child and Adolescent Psychiatry and Psychotherapy, Medical University of Vienna, Wien, Austria; ^4^Institute for Rare Diseases, Plovdiv, Bulgaria; ^5^Department of Social Medicine and Public Health, Faculty of Public Health, Medical University of Plovdiv, Plovdiv, Bulgaria; ^6^Université Toulouse Jean Jaurès, CERPPS, Toulouse, France; ^7^School of Nursing, Psychotherapy and Community Health, Dublin City University, Dublin, Ireland; ^8^The State Diagnostic and Counseling Center, Kópavogur, Iceland; ^9^Clinic of Child Psychiatry, University Hospital of Oulu, Oulu, Finland; ^10^Medical Faculty, PEDEGO Research Unit, Oulu University, Oulu, Finland; ^11^Faculty of Social Sciences, University of Tampere, Logopedics, Tampere, Finland; ^12^Finnish Institute for Health and Welfare, Helsinki, Finland; ^13^Research Center for Child Psychiatry, University of Turku, Turku, Finland; ^14^Division of Family Medicine, Department of Neurobiology, Care Sciences and Society, Stockholm, Karolinska Institute, Stockholm, Sweden; ^15^Finnish Association for Autism and Asperger's Syndrome, Helsinki, Finland; ^16^Department of Psychiatry, University of Helsinki, Helsinki, Finland; ^17^Faculty of Psychology, University of Warsaw, Warsaw, Poland; ^18^Instituto Nacional de Saúde Doutor Ricardo Jorge, Center for Biodiversity, Functional and Integrative Genomics, Lisbon, Portugal; ^19^‘Victor Babeş’ National Institute for Research and Development in Pathology and Biomedical Sciences, Timisoara, Romania; ^20^National Autistic Society, The Center for Autism, London, United Kingdom; ^21^Departamento de Personalidad, Evaluación y Tratamiento Psicológicos, INICO - Instituto Universitario de Integración en la Comunidad University of Salamanca, Salamanca, Spain; ^22^Departamento de Psicología Evolutiva y de la Educación, INICO - Instituto Universitario de Integración en la Comunidad University of Salamanca, Salamanca, Spain; ^23^Instituto de Salud Carlos III, Institute of Rare Diseases Research Madrid, Madrid, Spain; ^24^Kings College London, Institute of Psychiatry, Psychology and Neuroscience, London, United Kingdom; ^25^Department of Public Health, Aarhus University, Aarhus, Denmark; ^26^Lundbeck Foundation Initiative for Integrative Psychiatric Research, iPSYCH, Aarhus, Denmark; ^27^Department of Economics and Business, National Center for Register-Based Research, Aarhus University, Aarhus, Denmark

**Keywords:** autism spectrum disorder, adults, health, co-occurring conditions, services

## Abstract

The Autism Spectrum Disorders in the European Union (ASDEU) survey investigated the knowledge and health service experiences of users and providers to generate new hypotheses and scientific investigations that would contribute to improvement in health care for autistic adults. An online survey designed for autistic adults, carers of autistic adults, and professionals in adult services was translated into 11 languages and distributed electronically by organizations and in-country adult service facilities in 2017; 522 autistic adults, 442 carers, and 113 professionals provided answers to the health questions. Professionals, the majority in non-medical services, appeared to be poorly informed about whether certain co-occurring conditions were more frequent in autistic adults than typical adults—especially some medical conditions, suicide attempts, accidents, and pain. A minority of autistic adults reported preventive health behaviors such as routine health check-ups. The majority of users and providers expressed the desire to make health care services more user-friendly for autistic adults. Among the three groups, <20% of responders knew an organization or clinician which has developed a way to monitor health, and prevent poor health, that works well for adults on the autism spectrum. The results point to means for better management of co-occurring conditions associated with autism in adulthood in order to reduce hospital admissions and potential areas of improvement in health and social services for autistic adults. Specifically, efforts should be focused on (1) professionals' education on risks for co-occurring conditions in autistic adults; (2) promoting preventive health behaviors; (3) making services user-friendly for autistic adults and their families; and (4) encouraging knowledge of good local services.

## Introduction

Autism spectrum disorder is characterized by impairments in social communication, the presence of restricted and repetitive behaviors, and interests and sensory anomalies (1). The latest measurements in Europe and in the USA reveal a considerable rise in measured prevalence of autism in the last decades [0.48 to 3.13%, (2–7)].

### Psychiatric and Medical Co-occurring Conditions in Autistic Adults

Autistic people more frequently have co-occurring conditions in adolescence and adulthood than the general population [e.g., (8–23)]. The prevalence of co-occurring psychiatric conditions has been estimated to range between 34 and 94% among autistic adolescents and adults [e.g., (11, 13, 22)]. The most common psychiatric conditions identified are depression, anxiety, attention-deficit hyperactivity disorder (ADHD), and obsessive-compulsive disorder (9, 10, 16, 17, 24, 25). Autistic adults also have higher rates of unintentional injury, self-harm behavior, suicide attempts, or completed suicide, than non-autistic adults (10, 26–28). The prevalence of other health conditions is also reported to be high, ranging from 11 to 42% among autistic adolescents and adults (11), including infections, overweight, obesity, epilepsy, gastrointestinal (GI) disorders, sleep disorders, hypertension, allergies, and diabetes (10, 11, 29, 30). The prevalence of co-occurring conditions in autistic adults might be influenced by age, level of functioning, gender, education level, and cognitive functioning (16, 21, 31–33).

Co-occurring health/psychiatric co-occurring conditions and medical problems dramatically compromise the autistic adult's adaptive skills and quality of life. Indeed, co-occurring psychiatric conditions in autistic adults are associated with risk of poorer psychosocial and adaptive functioning outcomes, employment, and quality of life (13, 25, 34, 35). A striking example is depression in autistic adults which is associated with suicidal ideation and plans or attempts (36–39).

### Services for Managing Co-occurring Conditions in Autistic Adults

Many people with autism face challenges throughout their lifespan and most will rely on support from their families and communities throughout life. Due to the increasing number of autistic people entering adulthood and the high prevalence of co-occurring conditions in this population, there is an urgent need to assess health characteristics, use of the health services, and identify room for improvement and to promote better health care models. Research on health services for autistic adults is limited. In recent studies, autistic adults and their carers have reported difficulties in accessing preventive, treatment, and support services (33, 40–42) and poor knowledge among professionals of co-occurring mental health difficulties in autism (41). Also, health care providers perceive their own lack of knowledge concerning autistic adults' treatment and recognize the need to enhance their training on strategies for managing health conditions of autistic adults (43–45). Finally, autistic adolescents and adults are more likely to use emergency services compared with their typically developing peers [e.g., (30, 46)] and emergency services workers might be poorly trained on autistic people's specific needs (47). It has been recommended that (1) multidisciplinary health services should focus on prevention of the onset of co-occurring conditions (48); (2) coordinate care through time; (3) potentiate autism-specific training and competencies in people at all organizational levels involved in care of autistic adults (49); (4) identify local strategic plans and a designated local responsible lead over provision of adult autistic services; (5) include autistic people and families in decision-making and processes; and (6) encourage transparent processes for assessment of needs and screen for less clinically researched dangerous behaviors such as accidents with injuries, suicide attempts, self-harm behaviors, and being victim of abuse or violence (10, 27, 50).

## Aims and Objectives

The present study aims to explore the experiences and perceptions by autistic adults, carers of autistic adults and adult service professionals on psychiatric and medical co-occurring conditions and health behaviors in autistic adults. We used data from an online survey developed by the Autism Spectrum the Disorder in the European Union (ASDEU 2015–2018) network regarding a variety of topics in autistic adult services and care and was distributed in 11 European countries. Among other topics, the survey collected information on co-occurring conditions and reasons for medical contacts reported by autistic adults, carers, and professionals. Participants were also asked about positive and poor health behaviors, their views on ways to promote better health, and their knowledge of health services for autistic adults. The overall goal was to collect a large body of descriptive information on practices and experiences in autistic adults' health care in Europe that would generate new hypotheses and scientific investigations that would contribute to the development of best practices in autistic adult health care and support. Also, studies that investigate the autistic adults', carers', and providers' experience and perceptions when assessing health care services for autistic adults are extremely relevant as they provide an evidence base to inform national and international health and social care sector policies.

## Methods

### Survey Description

Health characteristics to include in the survey were selected based on literature reports of common health problems and concerns for autistic adults. Separate versions of the ASDEU online survey were developed for the three target groups: autistic adults diagnosed in adulthood; family/caregivers of autistic adults; and administrators/professionals/service providers for adults. The survey questions and answers were formulated in everyday language that might be understood among the three groups and in different countries. During survey development, professionals in autism services (diagnosis, treatment, services) and representatives from an autism advocacy organization were asked to review and comment on survey drafts. The online version for autistic adults was tested by an autistic adult who gave feedback on language. This paper reports the analysis of data from two sections of the survey: (1) background characteristics, including 12 question for the autistic adults, 12 for carers, and seven for professionals; (2) health characteristics in adults with autism spectrum, including nine question for autistic adults, 10 for carers, and nine for professionals (for the questions, see Supplementary Table 1).

### Survey Distribution

The lead site (Denmark) developed procedures on how best to distribute the survey. All 11 partners invited national or local autism organizations or service providers (public or private; including residential facilities, job training, and education programs) to voluntarily participate in the distribution and completion of the survey. These organizations also published the survey links in their e-newsletters, websites, or social media accounts inviting volunteers to participate. The link for completing the survey in its three versions (autistic adult, carer, and professional), was accessible starting in mid-February 2017 in three languages (English, Spanish, and Danish). By mid-September 2017, the three versions of the survey had been launched online in 11 languages [English, Spanish, Danish, French, Polish, Icelandic, German, Finnish, Italian, Romanian, Portuguese (professional version only)]. Data were frozen for analysis in December 2017. Participants were asked to give their informed consent electronically before starting the survey. Responders were instructed to choose the answer that best met their experience or knowledge. Answers were completely anonymous; hence, there was no linkage, if any, of respondents between groups (e.g., an autistic adult and his/her provider). Data were analyzed in aggregated form.

### Statistical Analysis

We received 2,009 completed or partially completed surveys (autistic adults: *n* = 667; carers of autistic adults: *n* = 591; professionals: *n* = 751). For the purpose of the present study, only demographic characteristics and responses specific to the health services were analyzed. The health section was completed by 1,077 responders, including 522 adult responders who provided responses to at least one question in the health and managing co-occurring conditions section; 442 carers who answered YES to the question “You should answer this section ONLY if you have experience in the last 2 years regarding the health of the autistic adult.”; and 113 professionals who answered YES to the question “Do you have professional knowledge of or current work experience in health conditions, health behaviors and medical contacts in autistic adults?”

Aggregated summary statistics (i.e., frequencies and percentages of responses to questions) were calculated for all questions. The frequencies reported in the section Results always refer to the number of responders for each question. Thus, sample size varies depending on the specific question.

One of the key objectives of our analyses was to assess if the prevalences of reported conditions by the adults and carers were simply higher, lower, or about the same as what we would expect based on rates of the conditions in the general population, realizing that the specific prevalence values based on self-report in our sample of volunteers could be biased. Since we did not have a general population comparison group in this study, we searched the literature for prevalence studies of the conditions in general population samples. It was not possible to find published rates that were directly comparable with our study data (e.g., similar age range, calendar time period, reporting period). Thus, the general population prevalence rates that we present for comparison with the adults' and carers' responses should be used with caution and only as a rough guide.

Where sample sizes permitted, we performed Chi-square tests (with Yates continuity corrections) on the affirmative answers to explore if (1) males vs. females and (2) medical vs. non-medical professionals differed in reporting co-occurring conditions (health profile of autistic adults' section; Supplementary Tables 2, 3). Due to the small sample size (*n* = 19), the responders that answered “Other/no answer” to the gender question were excluded from the gender analysis.

## Results

### Demographics

As shown in Supplementary Table 2, the majority of the responders were living in Denmark (33%, *n* = 368), France (15%, *n* = 163), Finland (12%, *n* = 127), Spain (11%, *n* = 120), Poland (8%, *n* = 88), Italy (8%, *n* = 86), and Iceland (6%, *n* = 67) and lived in cities that are not capital cities (70%; *n* = 753). Respondents were primarily women (autistic adults: 66%, *n* = 346; carers: 82%, *n* = 362; professionals: 68%, *n* = 77), while the cared-for autistic adults were mainly men (72%, *n* = 316). Almost half of the autistic adults (44%) were over 35 years of age whereas only 17% of the cared-for autistic adults were over 35.

About half of cared-for autistic adults had some level of independence (high level of independence, 9%, *n* = 38; some independence but need support, 39%, *n* = 173), whereas the other half required a high level of support (needs a high level of support in daily living, 35%, *n* = 155; needs high-level institution-like care, 17%, *n* = 76). The majority, 86% (*n* = 381) of carers were parents of autistic adults.

Most professionals (87%) worked in non-medical services (i.e., psychologist, teacher/pedagogue, social worker, physical or occupational therapist, nurse, teaching assistant/nursery assistant, mental health therapist, criminal justice) while the rest were in adult medical-type services (i.e., psychiatrist, general practitioner, medical specialist other than psychiatrist, other medical professional). The most commonly represented professional backgrounds were psychologists (35%, *n* = 39), teachers/pedagogues (11%, *n* = 12), and psychiatrists (11%, *n* = 12).

### Health and Managing Co-Occurring Conditions

#### Health Profile of Autistic Adults

**Figure 1:**
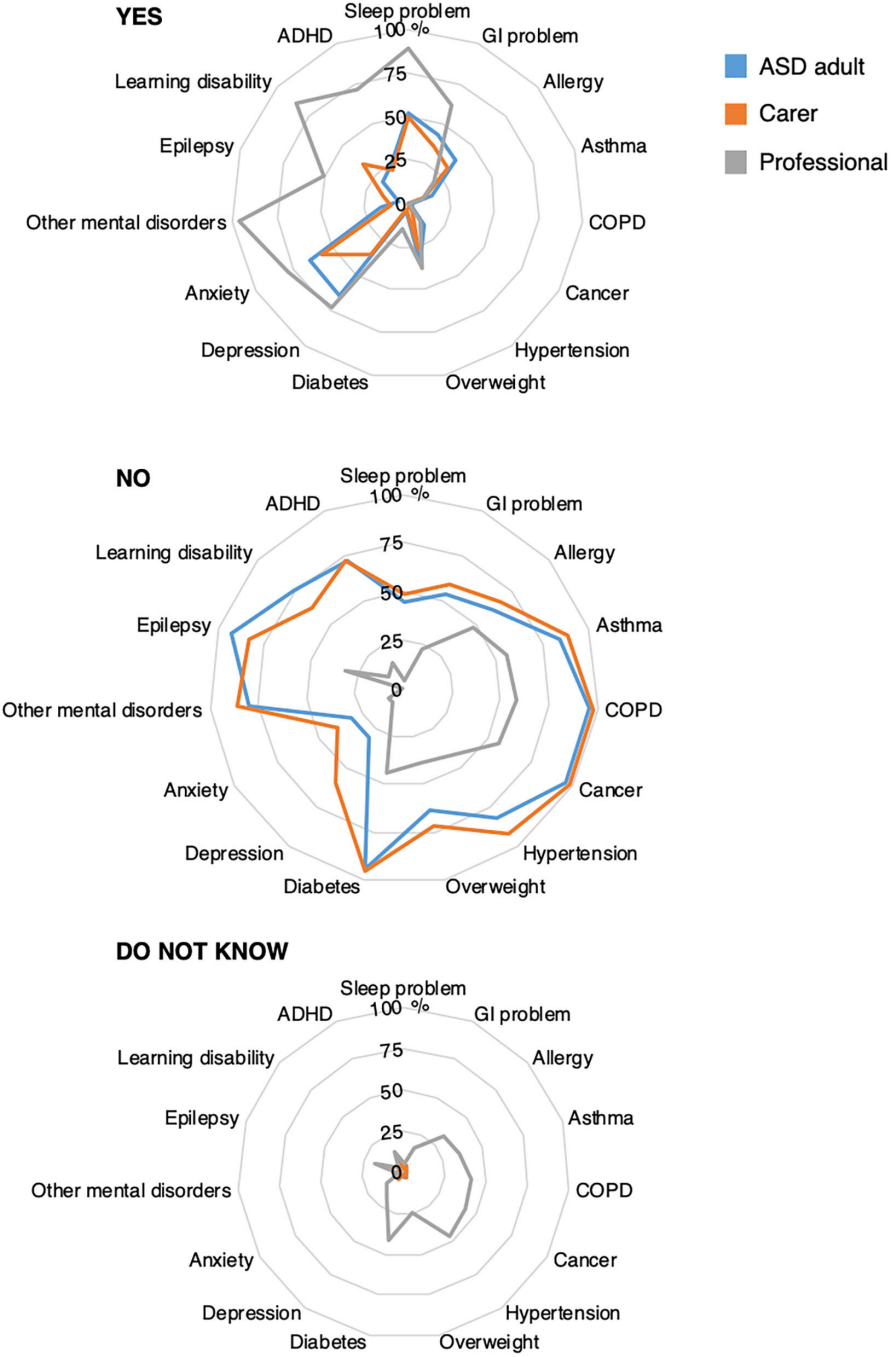
Health profile in autistic adult. GI, gastrointestinal; COPD, chronic obstructive pulmonary disease; ADHD, attention-deficit hyperactivity disorder. Radar chart plots of the answers “YES,” “NO,” and “Do not know” at the following questions: “Since you became an adult … have you been diagnosed by a doctor with:” (autistic adults, blue line); “In the last 2 years, has the autistic adult been diagnosed or treated by a doctor for:” (carers, orange line) and “… do you believe that the following conditions are more frequent in autistic adults, compared with adults not on the autism spectrum?” (Professionals, gray line). Values expressed as percentages. *N* autistic adults 521 to 522; *N* carers 437 to 440; *N* professionals: 113. Infection is not displayed in the figure since it is not a chronic condition.

As shown in Table 1, autistic adult responders (reporting diagnoses since their 18th birthday) and carers (reporting diagnoses in the cared for adults in the last 2 years) reported diagnoses of depression, anxiety, sleep problems, ADHD, learning disability, epilepsy, self-harm or injury, accidents with injuries, suicide attempts, and GI problems much more or somewhat more often than the published prevalence of these conditions in general adult populations. Allergy was reported by both autistic adults and cares in slightly higher rates than a published general population prevalence and asthma and cancer were reported at slightly higher rates by the autistic adults. In contrast, infection, COPD, hypertension, overweight, diabetes, and other mental or psychiatric conditions (and cancer and asthma by carers) were reported at similar or lower frequencies than reported in the published references. Among both autistic adult responders and carers, the most frequently reported co-occurring conditions were sleep problems, anxiety (>50%), and depression (65% by adult responders) (Table 1; Figure 1). Between 25 and 50% of adults or carers' (for their adults) reported a GI problem, allergies, overweight, self-harm/injury (carers), or learning disability (carers). Less than 5% of adults or carers reported chronic obstructive pulmonary disease (COPD), cancer, or diabetes.

**Table 1:** Health profile of autistic adults.

**Answer**	**Reported prevalence of the condition in general population adults (%)**	**Autistic adult**			**Carer**			**Professional**		
		**Yes**	**No**	**Do not know**	**Yes**	**No**	**Do not know**	**Yes, more frequent**	**No, not more frequent**	**Do not know**
Sleep problems	9.6^a^	267 (51.3)	235 (45.1)	19 (3.7)	220 (50.1)	212 (48.3)	7 (1.6)	101 (89.4)	5 (4.4)	7 (6.2)
GI problem	27.5^a^	225 (43.1)	281 (53.8)	16 (3.1)	158 (35.9)	259 (58.9)	23 (5.2)	69 (61.1)	26 (23.0)	18 (15.9)
Infection	72.5^a^	192 (36.9)	310 (59.5)	19 (3.7)	119 (27.1)	308 (70.0)	13 (3.0)	16 (14.2)	66 (58.4)	31 (27.4)
Allergy	28.7^a^	191 (36.6)	318 (60.9)	13 (2.5)	137 (31.3)	291 (66.4)	10 (2.3)	22 (19.5)	54 (47.8)	37 (32.7)
Asthma	10.3^a^	73 (14.0)	437 (83.9)	11 (2.1)	45 (10.3)	387 (88.6)	5 (1.1)	10 (8.9)	63 (55.8)	40 (35.4)
COPD	6.3^b^	11 (2.1)	500 (96.0)	10 (1.9)	2 (0.5)	429 (98.2)	6 (1.4)	1 (0.9)	66 (58.4)	46 (40.7)
Cancer	2.2^a^	16 (3.1)	498 (95.6)	7 (1.3)	3 (0.7)	429 (98.2)	5 (1.1)	2 (1.8)	63 (55.8)	48 (42.5)
Hypertension	15.6^a^/29^c^	80 (15.4)	427 (82.0)	14 (2.7)	21 (4.8)	400 (91.5)	16 (3.7)	13 (11.5)	47 (41.6)	53 (46.9)
Overweight	36.9 (men) −38 (women)^d^	175 (33.6)	334 (64.1)	12 (2.3)	116 (26.4)	317 (72.2)	6 (1.4)	42 (37.2)	44 (38.9)	27 (23.9)
Diabetes	4.3^a^	21 (4.0)	492 (94.4)	8 (1.5)	15 (3.4)	417 (95.2)	6 (1.4)	16 (14.2)	50 (44.3)	47 (41.6)
Depression	9.9^a^	340 (65.3)	162 (31.1)	19 (3.7)	155 (35.2)	262 (59.6)	23 (5.2)	89 (78.8)	10 (8.9)	14 (12.4)
Anxiety	9.1^a^	334 (64.1)	159 (30.5)	28 (5.4)	250 (57.1)	173 (39.5)	15 (3.4)	109 (96.5)	1 (0.9)	3 (2.7)
Other mental or psychiatric conditions	29.2 (lifetime)^e^	78 (15.0)	416 (79.9)	27 (5.2)	43 (9.8)	376 (86.0)	18 (4.1)	57 (50.4)	35 (31.0)	21 (18.6)
Epilepsy	0.7^a^	27 (5.2)	485 (92.9)	10 (1.9)	66 (15.1)	363 (82.9)	9 (2.1)	83 (73.5)	10 (8.9)	20 (17.7)
Accident	10.5 (men)/6.9 (women)^f^	N/A			90 (20.6)	344 (78.7)	3 (0.7)	59 (52.2)	23 (20.3)	31 (27.4)
Self-harm/injury	17.2 (adolescents) 13.4 (young adults)/5.5 (adults)^g^	N/A			145 (33.3)	280 (64.2)	11 (2.5)	97 (85.8)	7 (6.2)	9 (8.0)
Suicide attempt	0.3^a^	N/A			20 (4.6)	408 (93.6)	8 (1.8)	43 (38.1)	36 (31.9)	34 (30.1)
Learning disability	0.7^h^	98 (18.8)	393 (75.4)	30 (5.8)	148 (33.8)	275 (62.8)	15 (3.4)	97 (85.8)	11 (9.7)	5 (4.4)
ADHD	4.2^i^	122 (23.4)	377 (72.2)	23 (4.4)	90 (20.6)	318 (72.6)	30 (6.9)	81 (71.7)	17 (15.0)	15 (13.3)

*Values expressed as number of responders and frequencies (in parenthesis). N autistic adults: 521 to 522; N carers: 437 to 440; N professionals: 113. N/A, question not available for the correspondent group; GI, gastrointestinal; COPD, chronic obstructive pulmonary disease; ADHD, attention-deficit hyperactivity disorder. The questions were the following: autistic adult: “Since you became an adult (that is, after your 18 years birthday) have you been diagnosed by a doctor with:”; Carer: “In the last 2 years, has the autistic adult been diagnosed or treated by a doctor for:”; Professional: “Based on your professional knowledge or work experience, do you believe that the following conditions are more frequent in autistic adults, compared with adults not on the autism spectrum?.”*

a *Croen et al. (10)*;

b *Centers for Disease Control and Prevention (51)*;

c *Fryar et al. (52)*;

d *Ng et al. (29)*;

e *Steel et al. (53)*;

f *Saß et al. (50)*;

g *Swannell et al. (54)*;

h *Boyle et al. (55)*;

i *London and Landes (56)*.

At least 50% of professionals believed that sleep problems, GI problems, depression, anxiety, other mental or psychiatric conditions like schizophrenia, epilepsy, accident with injuries, self-harm or injury, learning disability, or ADHD were more frequent in autistic adults than non-autistic adults. Notably, however, a large proportion (>20%) of professionals did not know if allergy, asthma, COPD, cancers, hypertension, overweight, diabetes, accidents with injuries, or suicide attempts were more frequent in autistic adults than non-autistic adults (Figure 1; Table 1).

Although comparisons with published rates of the health conditions should be viewed with caution, it appears that for the conditions that were reported at higher or somewhat higher proportions by the autistic adults or carers [sleep problems, GI problem, depression, anxiety, epilepsy, accidents with injury, self-harm/injury, suicide attempts, learning disability (carers), or ADHD], all conditions except for suicide attempts were believed to occur more often in autistic than non-autistic adults by at least 50%, and typically >70%, of professionals (Table 1). Only 38% of professionals, however, believed that suicide attempts were more common in autistic than non-autistic adults.

**Figure 2:**
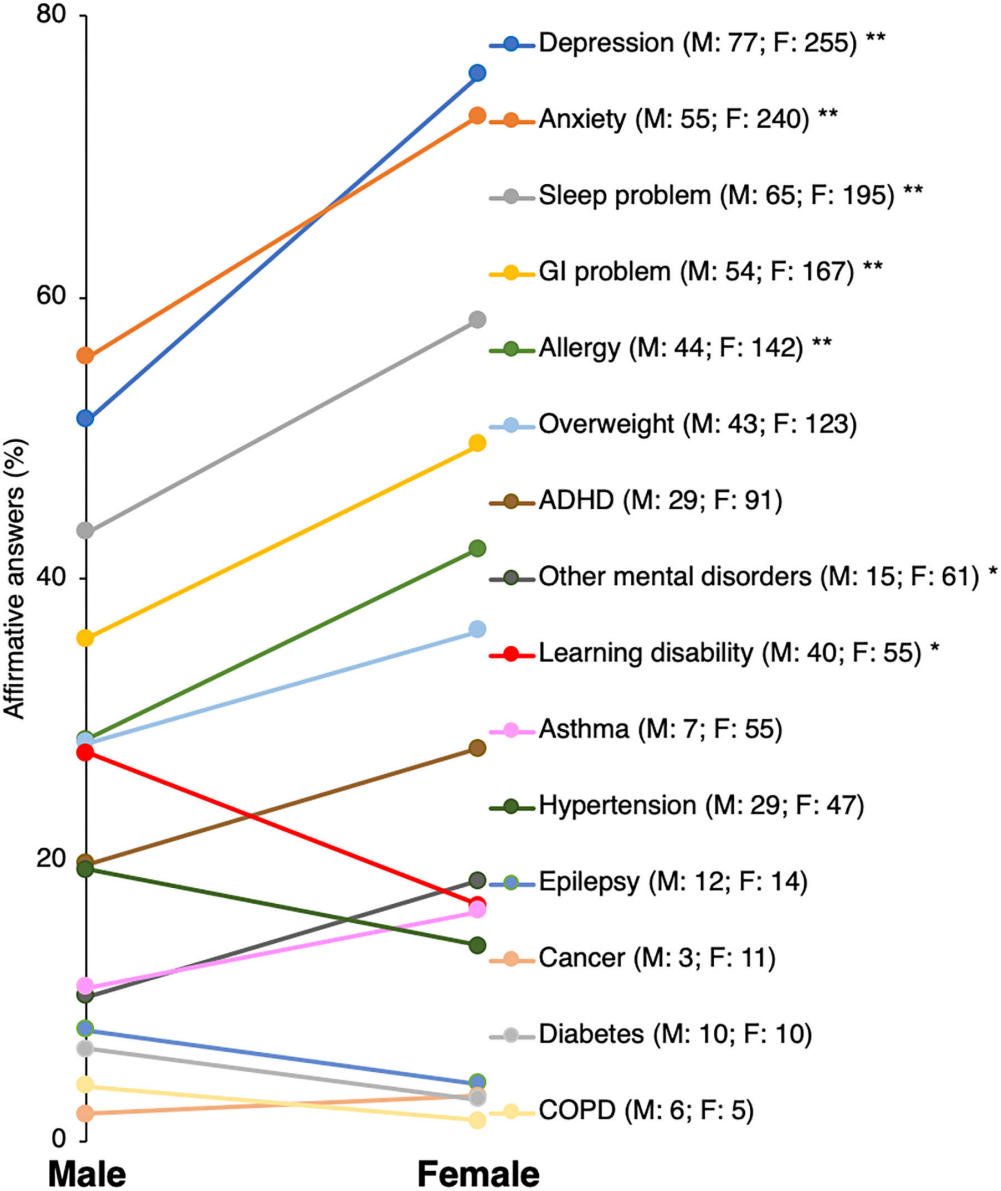
Differences in rates of chronic health conditions reported by autistic adult males and females. GI, gastrointestinal; COPD, chronic obstructive pulmonary disease; ADHD, attention-deficit hyperactivity disorder. *p < 0.05; **p < 0.005.

Chi-square tests for independence indicated a number of significant differences between autistic adult females and males in frequencies of chronic co-occurring conditions reported by autistic adult responders and carers. In particular, female autistic responders more frequently reported sleep problems, GI problems, allergy, depression, anxiety, and other mental or psychiatric conditions compared with males, while learning disability was reported more frequently by males (Figure 2; Supplementary Table 3). The only sex differences in diagnoses reported by carers were overweight and other mental or psychiatric conditions occurring more frequently in autistic females compared with males (Supplementary Table 3). Compared with non-medical professionals, medical professionals reported affirmative answers more often than non-medical professionals across all conditions except sleep problems although differences were infrequently statistically significant. Notably, only a minority of both medical and non-medical professionals believed that suicide attempts occurred more often in autistic than non-autistic adults (Supplementary Table 4).

#### Most Recent Hospital Contact (Inpatient, Outpatient, or Emergency Room Contacts)

**Figure 3:**
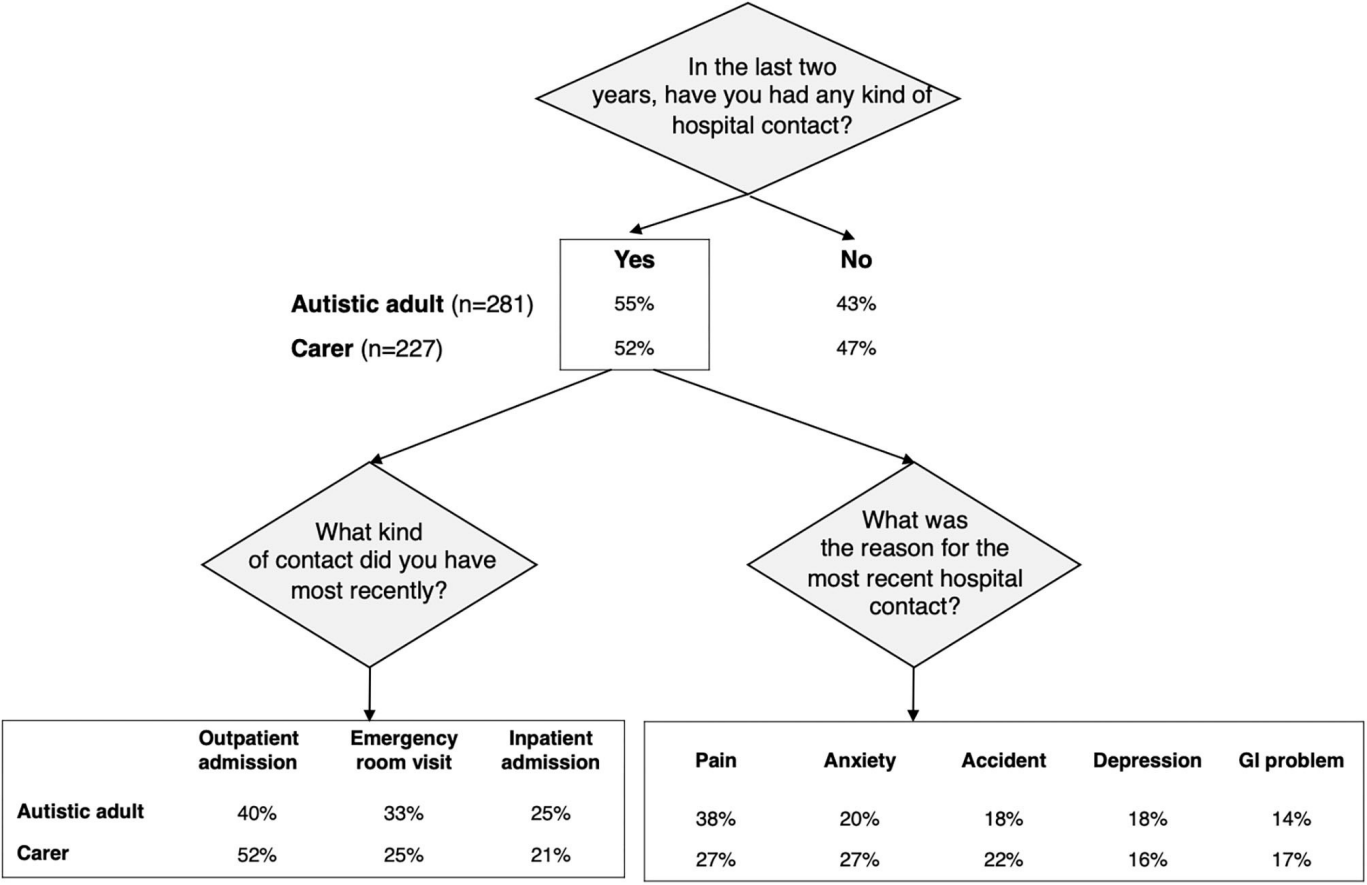
Characteristics of the autistic adult's most recent hospital contact in the last 2 years. GI, gastrointestinal. Flowchart summarizing percentages of positive responses given by the autistic adults and carers to the survey section related to the type/reason for most recent hospital contacts. “Do not know” answers are not displayed.

Autistic adults or carers of autistic adults were asked if they had any kind of hospital contact (in- or outpatient, emergency room) in the last 2 years. More than half of autistic adults and carers reported hospital contacts in the last 2 years (autistic adults: 55%, *n* = 281; carers: 52%, *n* = 227). The most frequently reported type of most recent contact was an outpatient admission (going to a medical clinic without an overnight stay; autistic adults: 40%, *n* = 115; carers: 52%, *n* = 117), whereas, somewhat fewer reported an emergency room visit (autistic adults: 33%, *n* = 96; carers: 25%, *n* = 60) or inpatient admission (going to the hospital with an overnight stay; autistic adults: 25%, *n* = 73; carers: 21%, *n* = 47) (Figure 3).

**Figure 4:**
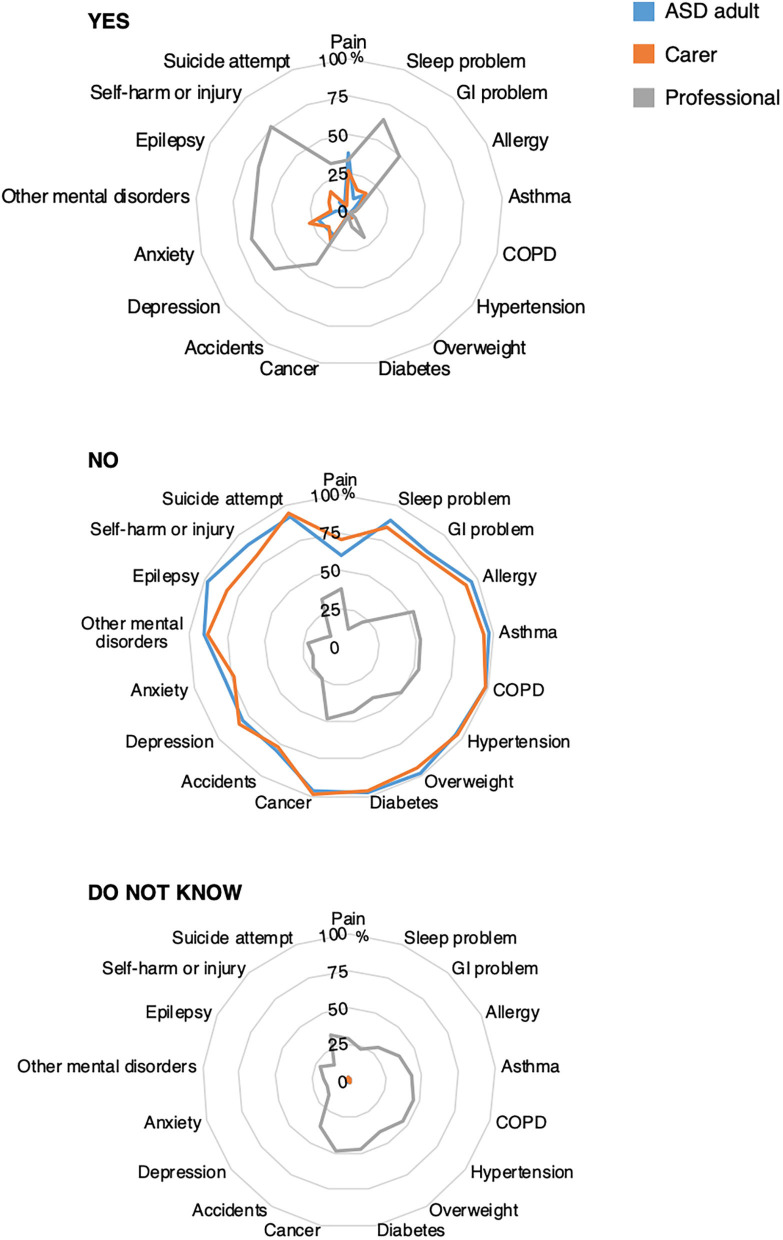
Reason for most recent hospital contact. GI, gastrointestinal; COPD, chronic obstructive pulmonary disease; ADHD, attention-deficit hyperactivity disorder. Radar chart plots of the answers “YES,” “NO,” and “Do not know” at the following questions: “What was the reason for the most recent hospital contact?” (autistic adults, blue line); “What was the reason for the adult's most recent hospital contact?” (carers, orange line) and “… Do you believe that the following reasons for hospital contacts are more frequent in autistic adults, compared with adults not on the autism spectrum?” (Professionals, gray line). Values expressed as percentages. *N* autistic adults: 273 to 274; *N* carers: 187 to 200; *N* professionals: 108. Infection is not displayed in the figure since it is not a chronic condition.

Six conditions were reported to be the reason for the most recent hospital contact by at least 10% of autistic adults: pain (38%), anxiety (20%), accident with injury (18%), depression (18%), GI problems (14%), and infection (12%). The same six conditions and four others were reported to be the reason for the most recent hospital contact by at least 10% of the carers: pain (27%), anxiety (27%), accident with injury (22%), infection (21%), self-harm/injury (18%), GI problems (17%), depression (16%,), sleep problems (15%), epilepsy (14%), and other mental or psychiatric conditions (12%). Notably, suicide attempt was the reason for the most recent contact by 7% of autistic adults and 4% of carers (Table 2; Figure 4). For the foregoing conditions, more than 50% of professionals believed that sleep problems, depression, anxiety, other mental or psychiatric conditions, epilepsy, and self-harm/injury were more frequent reasons for hospital contact by autistic adults than non-autistic adults but <50% believed that GI problems, infections, pain, or accidents with injuries would be more frequent reasons for hospital contact by autistic adults than non-autistic adults. Notably, only one out of three professionals believed that suicide attempt as a reason for hospital contact was more common among autistic than non-autistic adults (Table 2). Finally, for each condition, 15 to 49% of professionals responded that they did not know if a given condition was more often a reason for hospital contact among autistic than non-autistic adults (Table 2; Figure 4).

**Table 2:** Reason for most recent hospital contacts.

**Answer**	**Autistic adult**			**Carer**			**Professional**		
	**Yes**	**No**	**Do not know**	**Yes**	**No**	**Do not know**	**Yes, more frequent**	**No, not more frequent**	**Do not know**
Sleep problems	25 (9.2)	243 (89.0)	5 (1.8)	29 (15.1)	162 (84.4)	1 (0.5)	69 (63.9)	14 (13.0)	25 (23.2)
GI problem	39 (14.2)	230 (83.9)	5 (1.8)	32 (16.8)	155 (81.6)	3 (1.6)	52 (48.2)	23 (21.3)	33 (30.6)
Infection	32 (11.7)	239 (87.6)	2 (0.7)	42 (21.3)	153 (77.7)	2 (1.0)	12 (11.1)	51 (47.2)	45 (41.7)
Allergy	8 (2.9)	263 (96.3)	2 (0.7)	14 (7.3)	176 (91.7)	2 (1.0)	9 (8.3)	57 (52.8)	42 (38.9)
Asthma	6 (2.2)	265 (97.1)	2 (0.7)	9 (4.7)	180 (94.2)	2 (1.1)	5 (4.6)	57 (52.8)	46 (42.6)
COPD	1 (0.4)	269 (98.5)	3 (1.1)	1 (0.5)	187 (98.4)	2 (1.1)	1 (0.9)	57 (52.8)	50 (46.3)
Cancer	8 (2.9)	262 (96.0)	3 (1.1)	2 (1.1)	184 (98.4)	1 (0.5)	3 (2.8)	52 (48.2)	53 (49.1)
Hypertension	12 (4.4)	259 (94.9)	2 (0.7)	4 (2.1)	181 (96.3)	3 (1.6)	6 (5.6)	53 (49.1)	49 (45.4)
Overweight	3 (1.1)	268 (98.2)	2 (0.7)	9 (4.7)	179 (94.2)	2 (1.1)	21 (19.4)	43 (39.8)	44 (40.7)
Diabetes	6 (2.2)	265 (97.1)	2 (0.7)	7 (3.7)	181 (95.8)	1 (0.5)	10 (9.3)	47 (43.5)	51 (47.2)
Depression	49 (17.9)	222 (81.0)	3 (1.1)	31 (16.1)	161 (83.4)	1 (0.5)	66 (61.1)	24 (22.2)	18 (16.7)
Anxiety	55 (20.2)	215 (78.8)	3 (1.1)	53 (26.5)	145 (72.5)	2 (1.0)	71 (65.7)	21 (19.4)	16 (14.8)
Other mental or psychiatric conditions	24 (8.8)	246 (90.1)	3 (1.1)	22 (11.5)	168 (88.0)	1 (0.5)	66 (61.1)	23 (21.3)	19 (17.6)
Epilepsy	5 (1.8)	265 (97.1)	3 (1.1)	28 (14.4)	163 (84.0)	3 (1.6)	71 (65.7)	14 (13.0)	23 (21.3)
Pain	104 (38.1)	163 (59.7)	6 (2.2)	52 (26.7)	138 (70.8)	5 (2.6)	36 (33.3)	41 (38.0)	31 (28.7)
Accident	49 (18.0)	220 (80.6)	4 (1.5)	43 (21.9)	152 (77.6)	1 (0.5)	43 (39.8)	26 (24.1)	39 (36.1)
Self-harm/injury	22 (8.1)	247 (90.5)	4 (1.5)	35 (18.0)	158 (81.0)	2 (1.0)	81 (75.0)	11 (10.2)	16 (14.8)
Suicide attempt	18 (6.6)	251 (91.9)	4 (1.5)	8 (4.2)	177 (94.0)	4 (2.1)	36 (33.3)	36 (33.3)	36 (33.3)
Heart conditions	N/A	N/A	N/A	N/A	N/A	N/A	6 (5.6)	58 (53.7)	44 (40.7)
Lack of recognition of a health problem until it reached an advanced stage	29 (10.6)	241 (88.0)	4 (1.5)	34 (17.4)	157 (80.5)	4 (2.1)	70 (64.8)	12 (11.1)	26 (24.1)
Poor adherence to treatment for a previously diagnoses condition	53 (19.4)	214 (78.4)	6 (2.2)	25 (13.1)	164 (85.9)	2 (1.1)	55 (50.9)	20 (18.5)	33 (30.6)

*Values expressed as number of responders and frequencies (in parenthesis). N autistic adults: 273 to 274; N carers: 187 to 200; N professionals: 108. GI, gastrointestinal; COPD, chronic obstructive pulmonary disease; ADHD, attention-deficit hyperactivity disorder; N/A, question not available for the correspondent group. The questions were the following: autistic adult: “What was the reason for the most recent hospital contact?”; Carer: “What was the reason for the adult's most recent hospital contact?”; Professional: “Based on your professional knowledge or work experience, do you believe that the following reasons for hospital contacts (emergency room, inpatient or outpatient admission) are more frequent in autistic adults, compared with adults not on the autism spectrum?”*

Between 11 and 19% of adults and carers reported that the reason for their most recent hospital admission was due to poor adherence to treatment for a previously diagnosed condition or due to lack of recognition of a health problem until it reached an advanced stage. A majority of professionals (51 and 65%, respectively) believed that these reasons for hospital contact were more frequent in autistic adults than non-autistic adults (Figure 4; Table 2).

#### Health Behavior Profile

About 50% or more of adults and carers reported routine dental check-ups and regular physical activity; about 50% of carers also reported routine general health check-ups and about 50% of female autistic adults reported routine cervical smears. Less than 40% of adults and carers reported routine check-ups for vision, hearing, breast exams (for women), or sexual health. More than half of carers reported that the autistic adults had routine general health check-ups, while fewer autistic adults (34%) reported this kind of check-up. In comparison, 50 to 71% of professionals believed that each of these routine health check-ups were less frequent in autistic adults than non-autistic adults, while 10 to 32% of professionals did not know if each of these routine health check-ups were less frequent.

**Figure 5:**
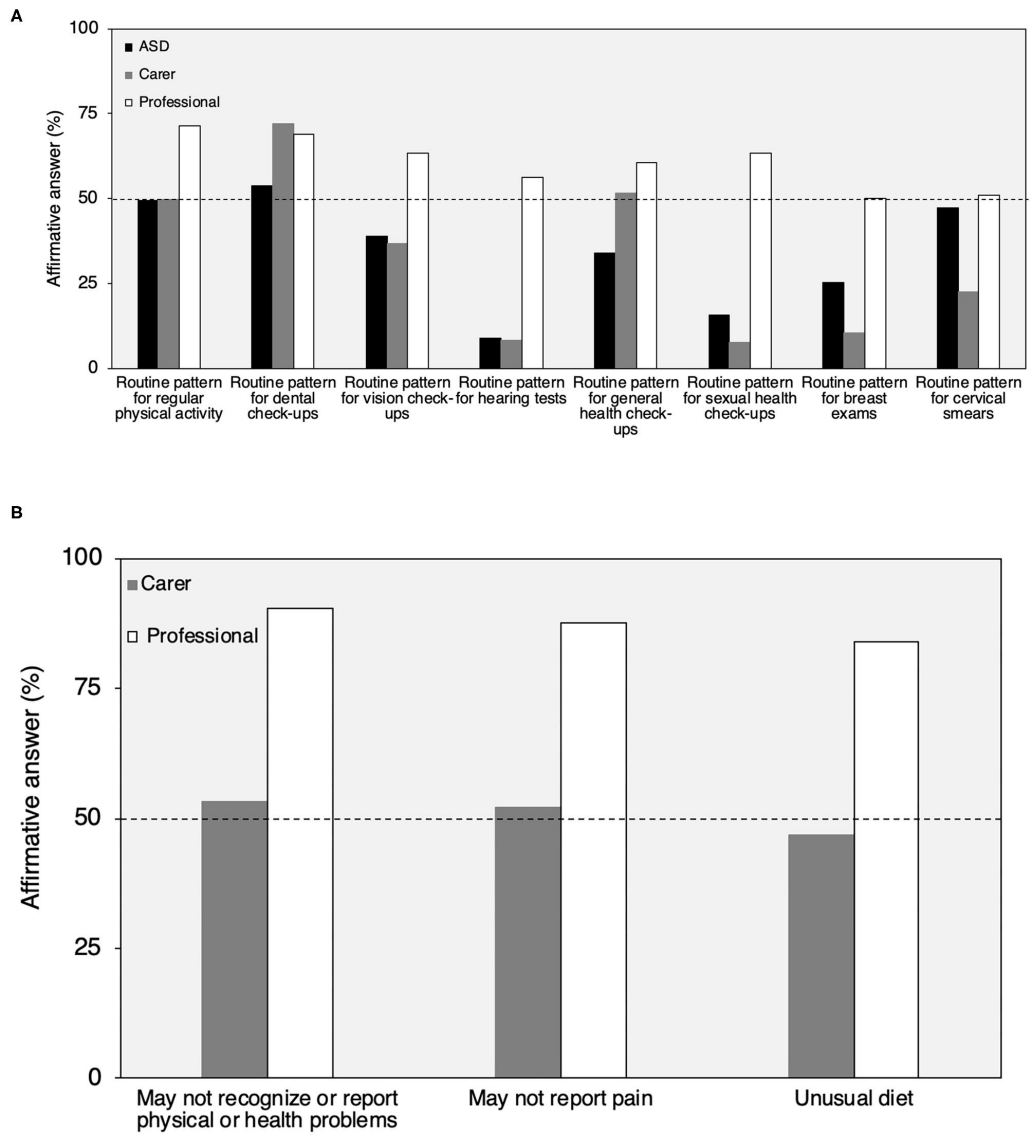
Health behavior profile reported by autistic adults, carers, and professionals **(A**,**B)**. Routine pattern for breast exams and cervical smears were collected only from autistic adult females (self-reported answers or reported by carers). *N* autistic adults: 515 to 342; *N* carers: 124 to 437; *N* professionals: 112 **(A)**. *N* carers: 437; *N* professionals: 113 **(B)**. In **(A)**, professionals answered YES if they believed the characteristic was LESS frequent in autistic than non-autistic adults. In **(B)**, they answered YES if they believed that the characteristic was MORE frequent in autistic than non-autistic adults.

About 50% of carers reported that the autistic adults they care for may not recognize or report physical or health problems or report pain or that the adult has an unusual diet. A large majority of professionals (84 to 90%) believed that these health characteristics, including not recognizing/reporting mental illness, were more frequent in autistic adults compared with adults not on the autism spectrum (Table 3; Figures 5A,B).

**Table 3:** Health behavior profile of autistic adults.

**Answer**	**Autistic adult**			**Carer**			**Professional**		
	**Yes**	**No**	**Do not know**	**Yes**	**No**	**Do not know**	**Yes, LESS frequent**	**No, not LESS frequent**	**Do not know**
Routine pattern for regular physical activity	255 (49.5)	245 (47.6)	15 (2.9)	217 (49.7)	214 (49.0)	6 (1.4)	80 (71.4)	20 (17.9)	12 (10.7)
Routine pattern for dental check-ups	277 (53.9)	228 (44.4)	9 (1.8)	314 (71.9)	114 (26.1)	9 (2.1)	77 (68.8)	21 (18.8)	14 (12.5)
Routine pattern for vision check-ups	200 (38.9)	304 (59.1)	10 (2.0)	160 (36.6)	265 (60.6)	12 (2.8)	71 (63.4)	21 (18.8)	20 (17.9)
Routine pattern for hearing tests	46 (9.0)	455 (88.5)	13 (2.5)	36 (8.2)	387 (88.6)	14 (3.2)	63 (56.3)	24 (21.4)	25 (22.3)
Routine pattern for general health check-ups	174 (33.9)	327 (63.6)	13 (2.5)	226 (51.7)	198 (45.3)	13 (3.0)	68 (60.7)	28 (25.0)	16 (14.3)
Routine pattern for sexual health check-ups	80 (15.6)	422 (82.1)	12 (2.3)	33 (7.6)	379 (86.7)	25 (5.7)	71 (63.4)	18 (16.1)	23 (20.5)
Routine pattern for breast exams*	86 (25.2)	248 (72.5)	8 (2.3)	13 (10.5)	104 (83.9)	7 (5.7)	56 (50.0)	20 (17.9)	36 (32.1)
Routine pattern for cervical smears*	162 (47.4)	171 (50.0)	9 (2.6)	28 (22.6)	92 (74.2)	4 (3.2)	57 (50.9)	22 (19.6)	33 (29.5)
							**Yes, MORE frequent**	**No, not MORE frequent**	**Do not know**
May not recognize/report physical or health problems	N/A	N/A	N/A	233 (53.3)	176 (40.3)	28 (6.4)	102 (90.3)	5 (4.4)	6 (5.3)
May not report pain	N/A	N/A	N/A	228 (52.2)	193 (44.2)	16 (3.7)	99 (87.6)	4 (3.5)	10 (8.8)
Unusual diet	N/A	N/A	N/A	205 (46.9)	224 (51.3)	8 (1.8)	95 (84.1)	11 (9.7)	7 (6.2)
May not recognize/report mental illness	N/A	N/A	N/A	N/A	N/A	N/A	100 (88.5)	6 (5.3)	7 (6.2)

Values expressed as number of responders and frequencies (in parenthesis). N autistic adults: 342 to 515; N carers: 124 to 437; N professionals: 112 to 113. N/A, question not available for the correspondent group. *Answers collected only from autistic adult females (self-reported answers or deception of carers). The questions were the following: autistic adult: “Do you have any of the following health characteristics?”; Carer: “In the last 2 years, has the autistic person had any of the following health characteristics?”; Professional, for the first eight characteristics (from top to bottom): “Based on your professional knowledge or work experience, do you believe that the following characteristics are LESS frequent in autistic adults, compared with adults without autism spectrum?”; for the last four characteristics (bottom-most): “Based on your professional knowledge or work experience, do you believe that the following behaviors are MORE frequent in autistic adults, compared with adults not on the autism spectrum.”

#### Top Considerations to Promote Better Health

The two top choices for ways to improve autistic adult health selected by both carers and professionals were to make health care services more user-friendly for autistic adults and improve primary care provider's awareness of health risks in autistic adults. Other choices often made by carers were to learn more about early warning signs of illness and poor health (#3), development of a “health passport” (#4) and implement regular wellness check-ups in autistic adults (#5). For professionals, other top choices were health risks education for adults and carers (#3), development of a health passport (#4), and learn more about early warning signs of illness and poor health (#5) (Table 4).

**Table 4:** Top considerations to promote better health of autistic adults selected by carers and professionals.

**Answer**	**Carer**	**Professional**
	***n* (%)**	***n* (%)**
Adjustments that make health care services more user-friendly for autistic adults	252 (57.0)	54 (47.8)
Improve primary care provider's awareness of health risks in autistic adults	161 (36.4)	48 (42.5)
Early warning signs of illness or poor health in autistic adults	144 (32.6)	33 (29.2)
Develop a person's “health profile” part of an “autism passport”	134 (30.3)	35 (31.0)
Regular wellness check-ups in autistic adults	131 (29.6)	22 (19.5)
Health risks education programs for autistic adults, their carers and partners	114 (25.8)	46 (40.7)
Health promotion approaches for autistic adults	81 (18.3)	32 (28.3)
Ways to help autistic adults follow health care recommendations	78 (17.6)	11 (9.7)
I do not think that I can make a choice	40 (9.0)	10 (8.8)

*Values expressed as number of responders and frequencies (in parenthesis); 433 (98%) carers and 107 (95%) professionals selected at least one option, of whom 59 carers and 13 professionals selected one option, 16 carers and two professionals selected two options, and 358 carers and 92 professionals selected three options. The questions were the following: Carer: “Thinking of the reasons for hospital contacts in autistic adults, select 3 options below that you think might be top considerations for research to promote better health and prevent hospital contacts in adults with autism”; Professional: “Select 3 options below that you think might be top considerations for research to promote better health and prevent hospital contacts in adults on the autism spectrum.”*

#### Do You Know of a Good Local Model of Service?

The health characteristic section of the survey concluded with a question asking the respondent if they knew of an organization or clinician, in their area or elsewhere in the country, which has a way to monitor health and prevent poor health conditions that works well for adults with autism spectrum. Among all three respondent groups, the proportion who answered “YES” was very low (autistic adults: 8%; carers: 16%; professionals: 18%).

## Discussion

Results of an online survey from 1,077 autistic adults, carers of autistic adults, and professionals in adult services from 11 European countries provide insights into self-reported health characteristics of autistic adults as well as professionals' knowledge of and perceptions of autistic adult health.

### Health Profile of Autistic Adults

Although the specific health condition prevalence values based on the survey could be biased, the survey results of self-reported co-occurring health conditions are consistent with the previously reported higher prevalence of co-occurring conditions (i.e., sleep and GI problems, depression, anxiety, epilepsy, learning disability, ADHD, and accidents with injuries, self-harm or injuries, and suicide attempts) in autistic adults compared with the general population (8, 10, 11, 14, 15, 20, 21, 23, 50, 55–57). In addition, the present results are consistent with previously reported higher rates of co-occurring conditions in autistic adult females (i.e., sleep problems, GI problems, allergy, depression, anxiety, and other mental or psychiatric conditions) compared with autistic adult males [e.g., (21, 24, 33)], except for learning disabilities which was reported more often in this study by autistic adult males (58). These data support the considerable burden of chronic health problems among autistic adults, and especially autistic females. This knowledge regarding gender differences in autistic adult health could be applied to the development of health promotion and awareness programs for autistic adults and carers, primary care provider education around health assessment and care management of autistic adults, and development of primary and secondary health prevention programs for adults with autism.

Among all health conditions, adult responders showed higher frequencies compared with carer adults (except for epilepsy and learning disabilities). However, these differences in the frequencies of the health conditions in the two groups of adults are not directly comparable. First, responses by adults and carers reflected different time windows for getting a diagnosis of health conditions since the adults were reporting diagnoses received any time after they became 18 years old, whereas the carers were reporting diagnosis received in the last 2 years. Second, adults tended to be older than the carer adults and therefore could have higher frequencies of disorders that tend to increase in occurrence with age.

Professionals were generally aware that certain co-occurring conditions—especially mental/psychiatric/neurological conditions—occur more often in autistic adults than in the general population. The one serious condition which was not recognized as more frequent in autistic than non-autistic adults by the majority of professionals was suicide attempts. This is particularly worrying since it has been observed in a large-scale clinical study that autistic people contemplate suicide more often (66%) than the general population (17%) or than patients with psychosis (59%) (36). Research on suicidality in the autism population is urgently needed to define tailored prevention strategies because risk factors in autistic people are different to those for the general population (59). Although some previous studies [e.g., (10)] have reported many conditions to be higher in autistic than non-autistic adults, i.e., allergies, asthma, pulmonary diseases, hypertension, obesity, and diabetes, only a minority of professionals in this study believed the latter conditions to be more common in autistic than non-autistic adults. Although professionals were first screened for their knowledge and experience in health and health care for autistic adults before they could proceed to the health question section of the survey, these results may be due to the fact that the professionals consisted mostly of non-medical specialists. Also, the medical specialists in the current sample were overwhelmingly mental health professionals. More striking were the rates of the answer “Do not know” by professionals for co-occurring conditions that are well-known to be very frequent in autism such as depression, other mental or psychiatric conditions, epilepsy, and ADHD.

Thus, there appear to be gaps in professionals' knowledge of health care risks among adults with autism. These results highlight the urgent need to train professionals, especially non-medical specialists, on potential health problems for which autistic adults may be at high risk to avoid delayed recognition of a health conditions, identify specific needs, and make effective decisions about care (60, 61). The National Institute of Health and Clinical Excellence [(62); https://www.nice.org.uk/guidance/CG142] gives guidance to health professionals on interventions and support for autistic adults. Ongoing career training on autism awareness and possible associations between autism and specific physical and mental or psychiatric conditions may be necessary since studies have shown that physicians who report having received professional training in autism still felt the need for further training to care effectively for autistic adults (44, 63, 64). As Lai et al. (15) suggested, the health assessment of autistic adults should embrace a holistic approach during clinical screenings, assessments, and treatments. Preparing professionals in the health care of autistic adults should be a top priority for policy makers in autism (44).

### Reasons for Most Recent Hospital Contact

More than a half of autistic adults and cared-for autistic adults reported that they had a hospital contact in the last 2 years and, for the majority, the contact was an outpatient admission which is in line with Jariwala-Parikh et al. (65).

The most frequent reasons for hospital contact due to co-occurring conditions reported by both groups were pain, anxiety, accident with injury, depression, GI problems, and infection. Notably, suicide attempt was the reported reason for the most recent contact by 7% of autistic adults and 4% of carers. Some medical issues (GI problems, infections, pain, or accidents with injuries) as common reasons for hospitalizations in autistic adults were recognized by less than a half of professionals. Although professionals claimed to have recent professional knowledge and experience in health and health care for autistic adults, this result could be due to the fact that they were mainly non-medical specialists. Furthermore, only 33% of professionals reported that suicide attempts may be a cause of hospital contact more often for autistic adults although increased risk for suicide or suicide attempts in autism has been consistently observed (10, 28). Finally, the relatively high proportions of professionals who responded that they did not know if a given condition was more often a reason for hospital contact among autistic than non-autistic adults was striking. These results further underscore the need for provider training in the health profile and risks in autistic adults, particularly for non-medical specialists and providers. The risk of suicide in autism is one condition for which health provider education may be warranted.

The majority of professionals had the perception that autistic adults are more likely than non-autistic adults to have hospital contacts because they show poor adherence to treatment for a previously diagnosed condition or due to lack of recognition of a health problem until it reached an advanced stage. Around 10 to 20% of autistic adults and carers reported these as the reasons for their most recent hospital contact. These results suggest that these modifiable reasons for a medical contact could be a prime target for preventive intervention.

### Health Behavior Profile of Autistic Adults

The most frequent positive health behaviors reported by autistic adults or carers were routine patterns of regular physical activity, general health check-ups, dental check-ups, or cervical smears (for females) while only a minority reported routine exams for vision, hearing, breast exams (for women), or sexual health. More than a half of professionals perceived that these behaviors were less likely to take place among the autistic adult population than non-autistic adults although 10 to 32% of professionals did not know if each of these routine health check-ups were less frequent. These results indicate there may be gaps in routine primary health care practices for autistic adults that warrant attention, not least because the shortfalls may contribute to the high burden of co-occurring health problems in autistic adults. Bruder et al. (44) reported that 32% of physicians believed that having a yearly health checkup is the most common medical condition/medical need of autistic adults.

Another challenge in autistic adult health care is reflected in reports by large proportions of carers and professionals in this study that autistic adults may not recognize or report physical or health problems, pain, or mental illness (reported only by professionals), and that autistic adults have an unusual diet. The difficulties in reporting health, physical and psychological/psychiatric conditions have been identified as communication challenges for autistic adults without intellectual disability [i.e., identify, differentiate, generalize, name, localize and describe symptoms and observations of inner experiences, communicating needs to health professionals, unusual language, non-verbal communication challenges, literal understanding of language, not offering important information if not directly enquired about, lack of initiative to report information on health status; (61, 66–69)], as well as consequences of challenging patient-provider interactions health services settings [i.e., stress caused by open questions; lack of time to think and respond; health professionals' failure to use accessible language; lack of written notes; failed to allow patients to communicate their needs in writing; (66, 67, 70–72)]. Reporting pain, for example, may be particularly challenging for autistic people because of a blunted physical presentation or heightened pain tolerance, and difficulties in communicating sensory sensitivity issues (61, 72). The NICE guidelines suggest the use of a concrete and structured approach when communicating health information to autistic adults that considers the use of written and visual supports. Communication with autistic adults and carers should be promoted using explicit rules, plain language (e.g., avoiding metaphors, ambiguities, and hypothetical situations), offering regular breaks, and integrating special interests (e.g., computer presentation of information) (62). Education of carers and health care providers of these hazards and development of methods to address the challenges in communicating health conditions would seem to be another important opportunity for health care improvement for autistic adults.

### Recommendations for Top Considerations to Promote Better Health

The top choices selected by both carers and professionals for research areas into ways to promote better health and prevent hospital contacts in autistic adults were make health care services more user user friendly for autistic adults, improve primary care provider's awareness of health risks in autistic adults learn more about early warning signs of illness and poor health, and develop a health passport. In addition, the professionals also selected, as an important area of research, a health risks education programs for autistic adults, carers, and partners. These choices are consistent with the study results overall which point to the primary need for adult, carer and provider education on health risks in autistic adults as well as on health characteristics and behaviors which may increase the risk for poor health outcomes. The top choice—make health care services more user friendly—is just as important, however, to ensure that the education and health monitoring programs can be successfully implemented with autistic adult patients.

### Do You Know of a Good Local Model of Service?

Knowledge of organizations or clinicians which provided a way to monitor health and prevent poor health conditions that works well for autistic adults was extremely low among all respondent groups. This result underscores the many gaps in knowledge and awareness and challenges in health care delivery for autistic adults evident elsewhere in the study results, particularly in the respondents' selection of “adjustments that make health care services more user friendly for autistic adults” as a top consideration for better health promotion for autistic adults. Since much of the burden of care and reduced quality of life reported for the autistic adult population stems from mental and physical health problems (13, 25, 34, 35) the possible lack of good health care service models for autistic adults is a critical gap. However, the lack of knowledge of good service models may also point to an education and information gap, rather than a lack of good models per se. Both avenues are important components to be considered in future work in autistic adult health care service development. The NICE guidelines recommend that local care pathways are understandable, accessible, and acceptable for autistic adults, families, and professionals. It is also recommended that relevant professionals know the local autism care pathway and the way to access services. Information on local pathways should consider the person's knowledge and understanding of autism and its care and be appropriate to the local communities (62).

### Strengths and Limitations

The strengths of the present work are mainly the large sample size, particularly from individuals with autism and carers, and availability of answers from three respondent groups (autistic adults; carers; professionals), which is rarely seen in the literature.

The ASDEU survey was not distributed to a planned, predefined epidemiological study population. Instead, links to the survey were widely distributed online and *via* social media and inviting volunteers to participate. As responses by the volunteers were anonymous, we have no way of confirming the autism status of the adults or the carers' adults or the credentials of the professionals. We also have no way of detecting whether an autistic adult or carer of an autistic adult recruited their family member to answer the survey which would limit the degree of independence between responses. Thus, results from the study should be interpreted with caution since data were not collected using a rigorous scientific frame. It is plausible that potential selection biases in the recruitment (e.g., people with internet access, with contacts with local associations, and motivated to voluntarily participate) could impact the results, making comparisons between the respondent groups or to other studies invalid. Specific results should be replicated using well-powered and scientifically designed studies.

As there are no ongoing population-based assessments of demographic characteristics of autistic adults in the EU, the extent to which our study sample might differ from a true, representative sample is unknown. Most respondents were females even among autistic adults, which is common for online surveys (73) but experiences reported by autistic males may be under-represented. The expected male/female of 3:1 ratio in autism was not represented by our autistic adults' sample, whereas the cared-for autistic adults were more often males and more in line with the expected gender ratio in autism.

Responses across the autistic adult and carer respondent groups may not be directly comparable due to differences in age and level of independence of the autistic adults. Also, a potential bias may consist of answers from autistic adults with different levels of independence examined together. The single largest professional category among professionals was psychologist and the professional group was mainly represented by professionals working in non-medical services. This aspect has to be carefully considered for the interpretation of the professionals' answers, since medical services professionals are more closely involved in medical concerns of autistic adults. The single largest group of respondents lived in Denmark which may skew some results, but it is difficult to determine which results would be markedly impacted and in what direction. Responses by adults and carers reflected different time windows for getting a diagnosis of health conditions and the age differences between the autistic adult responders and the adults of the carer adults could impact the frequencies in these groups of disorders that tend to increase in occurrence with age. We did not collect data on the cultural background and lifestyle habits of the responders which may play a role in determining their health. Also, professionals' responses are based on their professional knowledge and experiences while users' responses were limited to their own personal experiences of services.

Another limitation was that the adult version of the survey did not provide an opportunity for autistic adults to make “top considerations to promote better health of autistic adults.” Future surveys should seek to explore also their opinions.

## Conclusion

The ASDEU survey distributed in 11 European counties provides several insights for designing future systematic studies and improving specific topic areas in services dedicated to autistic adults, including health. These survey data are strengthened by the fact that they derive from what is actually experienced by users and providers. The data provide inputs to detect best practices for the management of co-occurring conditions associated with autism in adulthood in order to reduce hospital admissions, and the development of quality standards for health and social services for autistic adults. This and similar studies are necessary to provide an evidence base around health care involving autistic adults and their carers and professionals to inform policy decisions.

From the results, it would appear that professionals may need better training and education on the prevalence and management of medical and psychiatric conditions in autistic adults, in order to be better partners with their autistic adult patients and families in managing specific health needs. Although there were gaps in knowledge of specific diagnoses, most concerning were gaps in awareness of such characteristics as risk for suicide, accidents with injuries, and pain. Professionals should be also aware that gender may play an important role in the occurrence of many conditions with higher rates in females for many conditions. Local care pathways should pay particular attention to promote access to health services for autistic women (62).

Second, it also appears crucial to promote better awareness of health risks, health education and good health behaviors among autistic adults and their carers. It would also be important to raise awareness of behaviors which challenge carers' and providers' ability to detect emerging or established health problems, such as autistic adults not recognizing or reporting physical or health problems, mental illness, or pain.

Third, all groups expressed the need to develop more user-friendly services for autistic adults, to train service providers on health risks in autistic adults and early warning signs of illness and poor health, and to develop a health passport containing the health information and needs of the autistic adult. It is also crucial to involve both users and providers in identifying the top priorities in research to promote better health of autistic adults.

Finally, it appears that future research should include the experiences and perceptions of autistic adults, families and professionals when considering autistic adult services' needs. All three perspectives are needed to fully understand the diverse services needs of members of the autism community and their carers.

## Data Availability Statement

The datasets presented in this article are not readily available because we can only share aggregated data (not individual level), and any data based on small sample size (<5) may not be shared as an extra data privacy precaution. Requests to access the datasets should be directed to Maria Luisa Scattoni, marialuisa.scattoni@iss.it; Diana Schendel, diana.schendel@ph.au.dk.

## Ethics Statement

The studies involving human participants were reviewed and approved by each ASDEU site that obtained local ethical approval as needed before distributing the survey in their country. All procedures in studies involving human participants were in accordance with the ethical standards of the institutional and/or national research committee and with the 1964 Helsinki declaration and its later amendments or comparable ethical standards. The patients/participants provided their written informed consent to participate in this study.

## Author Contributions

Formal analysis: MLS, TS, MM, DS, and AC. Writing—original draft preparation: MM, MLS, and DS. Funding acquisition for the survey dissemination and data collection and analysis (DGSANCO). Principal investigator: MP and MLS. Funding acquisition for the Italian participation at the survey, data analysis and writing (‘Osservatorio Italiano per il monitoraggio dei disturbi dello spettro autistico’ and ‘I disturbi dello spettro autistico: attività previste dal decreto ministeriale del 30.12.2016’). All authors contributed to the conceptualization, investigation, and writing—review and editing. All authors have read and agreed to the published version of the manuscript.

## Conflict of Interest

The authors declare that the research was conducted in the absence of any commercial or financial relationships that could be construed as a potential conflict of interest.
