# Supplementary material for: Autistic Adult Health and Professional Perceptions of It: Evidence From the ASDEU Project
Source: Front Psychiatry. 2021 May 28;12:614102. doi: 10.3389/fpsyt.2021.614102 (PMC8193054; doi:10.3389/fpsyt.2021.614102)
Supplement: Supplementary file 1 [file Table_1.DOCX]

**Table S1**

*Survey’s questions and answers for autistic adults, carers and professionals*

| Question autistic adult | Question carer | Question professional | Answer autistic adult | Answer carer | Answer professional |
| --- | --- | --- | --- | --- | --- |
| Demographic characteristics | | | | | |
| N/A | N/A | Thinking of your work history, what job or career title fits you best? | N/A | N/A | General practitioner |
|  |  |  |  |  | Psychiatrist |
|  |  |  |  |  | Medical specialist, other than psychiatrist |
|  |  |  |  |  | Nurse |
|  |  |  |  |  | Other medical professional |
|  |  |  |  |  | Psychologist |
|  |  |  |  |  | Social worker |
|  |  |  |  |  | Teacher/pedagogue |
|  |  |  |  |  | Teaching assistant/nursery assistant |
|  |  |  |  |  | Mental health therapist |
|  |  |  |  |  | Physical or occupational therapist |
|  |  |  |  |  | I work in the area of criminal justice (e.g., police, courts, legal advocate) |
|  |  |  |  |  | Other, please specify |
| N/A | N/A | Thinking of your work history, how many years in total have you been in jobs in adult services and care (social, medical, or other services) | N/A | N/A | < 1 year |
|  |  |  |  |  | 1-2 years |
|  |  |  |  |  | 3-5 years |
|  |  |  |  |  | 6-10 years |
|  |  |  |  |  | > 10 years |
| What is your gender? | What is your gender? | What is your gender? | Male | Male | Male |
|  |  |  | Female | Female | Female |
|  |  |  | Other/no answer | Other/no answer | Other/no answer |
| How old are you? | How old are you? | N/A | 18-25 | 18-25 | 18-25 |
|  |  |  | 26-35 | 26-35 | 26-35 |
|  |  |  | 36-45 | 36-45 | 36-45 |
|  |  |  | 46-55 | 46-55 | 46-55 |
|  |  |  | 56-64 | 56-64 | 56-64 |
|  |  |  | > 65 | > 65 | > 65 |
| N/A | How many years of education did you complete? | N/A | N/A | < 10 years | N/A |
|  |  |  |  | 10-12 years |  |
|  |  |  |  | 13-16 years |  |
|  |  |  |  | > 16 years |  |
| Are you going to a school, a home school or in an education program now? | N/A | N/A | Yes, full time | N/A | N/A |
|  |  |  | Yes, part time |  |  |
|  |  |  | No |  |  |
| If no, do you remember your age when you finished your education? | N/A | N/A | Yes | N/A | N/A |
|  |  |  | No |  |  |
| How old were you when you finished your education? | N/A | N/A | _______ | N/A | N/A |
| If yes, what kind of education are you in now? | N/A | N/A | Primary level school | N/A | N/A |
|  |  |  | Secondary level school (for example high school or gymnasium) |  |  |
|  |  |  | Technical, vocational or job training school |  |  |
|  |  |  | College or university |  |  |
| What kind of education were you in when you finished your education: | N/A | N/A | Secondary - level school (for example high school or gymnasium) | N/A | N/A |
|  |  |  | Technical, vocational or job training school |  |  |
|  |  |  | College or university |  |  |
|  |  |  | Don't know |  |  |
|  |  |  | Secondary - level school (for example high school or gymnasium) |  |  |
| Are you: | What is your current employment status now? Pick the answer that fits you best. | N/A | N/A | Student | N/A |
|  |  |  |  | Unemployed |  |
|  |  |  |  | Employed (part time or full time) |  |
|  |  |  |  | Self-employed |  |
|  |  |  |  | Retired |  |
|  |  |  |  | Volunteer |  |
| If you are unemployed, is it because: | N/A | N/A | You are a student | N/A | N/A |
|  |  |  | You are retired |  |  |
|  |  |  | You are looking for a job |  |  |
|  |  |  | You believe that you cannot find a job |  |  |
|  |  |  | You have a disability that prevents you from having a job |  |  |
|  |  |  | Other, please specify |  |  |
| What country do you live in? | What country do you live in? | What country do you work in? | Austria | Austria | Austria |
|  |  |  | Belgium | Belgium | Belgium |
|  |  |  | Bulgaria | Bulgaria | Bulgaria |
|  |  |  | Denmark | Denmark | Denmark |
|  |  |  | England | England | England |
|  |  |  | Finland | Finland | Finland |
|  |  |  | France | France | France |
|  |  |  | Iceland | Iceland | Iceland |
|  |  |  | Italy | Italy | Italy |
|  |  |  | Northern Ireland | Northern Ireland | Northern Ireland |
|  |  |  | Poland | Poland | Poland |
|  |  |  | Portugal | Portugal | Portugal |
|  |  |  | Republic of Ireland | Republic of Ireland | Republic of Ireland |
|  |  |  | Romania | Romania | Romania |
|  |  |  | Scotland | Scotland | Scotland |
|  |  |  | Spain | Spain | Spain |
|  |  |  | Wales | Wales | Wales |
|  |  |  | Other, specify | Other, specify | Other, specify |
| N/A | What is the autistic adult’s gender? | N/A | N/A | Male | N/A |
|  |  |  |  | Female |  |
|  |  |  |  | Other/no answer |  |
| N/A | How old is the autistic adult? | N/A | N/A | 18-25 | N/A |
|  |  |  |  | 26-35 |  |
|  |  |  |  | 36-45 |  |
|  |  |  |  | 46-55 |  |
|  |  |  |  | 56-64 |  |
|  |  |  |  | > 65 |  |
| N/A | How many years have you known the autistic adult? | N/A | N/A | Less than 1 year | N/A |
|  |  |  |  | 1-5 years |  |
|  |  |  |  | 5-10 years |  |
|  |  |  |  | More than 10 years but not the adult's whole life |  |
|  |  |  |  | The adult's whole life |  |
| N/A | How are you related to the autistic adult? | N/A | N/A | Parent | N/A |
|  |  |  |  | Other family member related by blood (not a parent, but for example a child, cousin, grand parent, uncle etc.) |  |
|  |  |  |  | Spouse or partner |  |
|  |  |  |  | A carer, but not a family member, spouse or partner |  |
| Where do you live now? | Where is the autistic adult living now? | Thinking of your current job location where is it located? | Capital city | Capital city | Capital city |
|  |  |  | Other than a capital city | Other than a capital city | Other than a capital city |
| How many people live in the community where you live now? (Answer the best you can) | How many people live in the community where the autistic adult lives now? Answer as best you can | How many people live in the community where your current job is located? | < 1.000 people | < 1.000 people | < 1.000 people |
|  |  |  | 1.000-20.000 people | 1.000-20.000 people | 1.000-20.000 people |
|  |  |  | 20.000-100.000 people | 20.000-100.000 people | 20.000-100.000 people |
|  |  |  | 100.000-1.000.000 | 100.000-1.000.000 | 100.000-1.000.000 |
|  |  |  | > 1.000.000 | > 1.000.000 | > 1.000.000 |
|  |  |  | Don't Know | Don't Know | Don't Know |
| N/A | Pick the description that best fits the autistic adult. | N/A | N/A | Has a high level of independence | N/A |
|  |  |  |  | Has some independence but needs support |  |
|  |  |  |  | Needs a high level of support in daily living |  |
|  |  |  |  | Needs high level institution-like care |  |
| N/A | N/A | The experience and knowledge about services for adults that you have from your current job is: | N/A | N/A | Most closely connected to your current job location (e.g., capital city or small town) |
|  |  |  |  |  | Most closely connected to your current job location and a wider area (e.g., the region or state where your job is located) |
|  |  |  |  |  | Most closely connected to the whole country |
| Health in adults with autism spectrum | | | | | |
| You should answer this section ONLY if you have experience in the last 2 years regarding the health of the autistic adult. | N/A | Do you have professional knowledge of or current work experience in health conditions, health behaviors and medical contacts in autistic adults? | N/A | Yes, I have experience in the last 2 years with the health of the autistic adult. | Yes |
|  |  |  |  | No, I don’t have experience in the last 2 years with the health of the autistic adult | No |
| Since you became an adult (that is, after your 18 years birthday) have you been diagnosed by a doctor with: (Yes; No; Don’t know) | In the last 2 years, has the autistic adult been diagnosed or treated by a doctor for: (Yes; No; Don’t know) | Based on your professional knowledge or work experience, do you believe that the following conditions are more frequent in autistic adults, compared to adults not on the autism spectrum? (Yes, more frequent; No, not more frequent; Don’t know) | Sleep problems | Sleep problems | Sleep problems |
|  |  |  | Stomach or digestion problems | Stomach or digestion problems | Stomach or digestion problems |
|  |  |  | Infections | Infections | Infections |
|  |  |  | Allergies | Allergies | Allergies |
|  |  |  | Asthma | Asthma | Asthma |
|  |  |  | Chronic obstructive pulmonary disease | Chronic obstructive pulmonary disease | Chronic obstructive pulmonary disease |
|  |  |  | Cancers | Cancers | Cancers |
|  |  |  | High blood pressure | High blood pressure | High blood pressure |
|  |  |  | Overweight | Overweight | Overweight |
|  |  |  | Diabetes | Diabetes | Diabetes |
|  |  |  | N/A | N/A | Vision problems |
|  |  |  | N/A | N/A | Hearing impairment |
|  |  |  | Depression | Depression | Depression |
|  |  |  | N/A | N/A | Stress |
|  |  |  | Anxiety | Anxiety | Anxiety |
|  |  |  | Mental disorders, like schizophrenia | Mental disorders, like schizophrenia | Other mental or psychiatric disorders, like schizophrenia |
|  |  |  | Epilepsy | Epilepsy | Epilepsy |
|  |  |  | Learning disability | Learning disability | Learning disability |
|  |  |  | ADHD | ADHD | ADHD |
| N/A | In the last 2 years, has the autistic person had: (Yes; No; Don’t know) |  | N/A | Accidents with injuries | Accidents with injuries |
|  |  |  |  | Self-harm or self-injury | Self-harm or injury |
|  |  |  |  | Suicide or suicide attempt |  |
| Do you have any of the following health characteristic? (Yes; No; Don’t know) | In the last 2 years, has the autistic person had any of the following health characteristics? (Yes; No; Don’t know) | Based on your professional knowledge or work experience, do you believe that the following behaviors are more frequent in autistic adults, compared to adults not on the autism spectrum? (Yes, more frequent; No, not more frequent; Don’t know) | N/A | May not recognize or report physical or health problems | May not recognize or report physical or health problems |
|  |  |  | N/A | N/A | May not recognize or report mental illness |
|  |  |  | N/A | May not report pain | May not report pain |
|  |  |  | N/A | Unusual diet | Unusual diet |
|  |  | Based on your professional knowledge or work experience, do you believe that the following characteristics are less frequent in autistic adults, compared to adults without autism spectrum? (Yes, more frequent; No, not more frequent; Don’t know) | You follow a kind of routine pattern for regular physical activity | Regular physical activity | Regular physical activity |
|  |  |  | You follow a kind of routine pattern for dental check-ups | Routine dental check-ups | Routine dental check-ups |
|  |  |  | You follow a kind of routine pattern for vision check-ups | Routine vision check-ups | Routine vision check-ups |
|  |  |  | You follow a kind of routine pattern for hearing tests | Hearing tests | Hearing tests |
|  |  |  | You follow a kind of routine pattern for general health check-ups | Routine general health check-ups | Routine general health check-ups |
|  |  |  | You follow a kind of routine pattern for sexual health check-ups | Sexual health check-ups | Sexual health check-ups |
|  |  |  | You follow a kind of routine pattern for breast exams (female) | Routine breast exams for women | Routine breast exams for women |
|  |  |  | You follow a kind of routine pattern for cervical smears (female) | Routine cervical smears for women | Routine cervical smears for women |
| A hospital contact can be an emergency room visit, or going to the hospital that included an overnight stay (called an inpatient admission), or going to a medical clinic without an overnight stay (called an outpatient admission). In the last 2 years have you had any kind of hospital contact? | A hospital contact can be an emergency room visit, or going to the hospital that included an overnight stay (called an inpatient admission), or going to a medical clinic without an overnight stay (called an outpatient admission). In the last 2 years has the autistic adult had any kind of hospital contact? | N/A | Yes | Yes | N/A |
|  |  |  | No | No |  |
|  |  |  | Don’t know | Don’t know |  |
| If yes, what kind of contact did you have most recently? | If yes, what kind of contact did the adult have most recently? | N/A | Emergency room visit | Emergency room visit | N/A |
|  |  |  | Inpatient admission (going to the hospital with an overnight stay) | Inpatient admission (going to the hospital with an overnight stay) |  |
|  |  |  | Outpatient admission (going to a medical clinic without an overnight stay) | Outpatient admission (going to a medical clinic without an overnight stay) |  |
|  |  |  | Don’t know | Don’t know |  |
| What was the reason for your most recent hospital contact? (Pick all that apply) (Yes; No; Don’t know) | What was the reason for the adult’s most recent hospital contact? (Check all that apply) | Based on your professional knowledge or work experience, do you believe that the following reasons for hospital contacts (emergency room, inpatient or outpatient admission) are more frequent in autistic adults, compared to adults not on the autism spectrum? | Pain | Pain | Pain |
|  |  |  | N/A | N/A | Heart conditions |
|  |  |  | Sleep problems | Sleep problems | Sleep problems |
|  |  |  | Stomach or digestion problems | Stomach or digestion problems | Stomach or digestion problems |
|  |  |  | Infections | Infections | Infections |
|  |  |  | Allergies | Allergies | Allergies |
|  |  |  | Asthma | Asthma | Asthma |
|  |  |  | Chronic obstructive pulmonary disease | Chronic obstructive pulmonary disease | Chronic obstructive pulmonary disease |
|  |  |  | High blood pressure | High blood pressure | High blood pressure |
|  |  |  | Overweight | Overweight | Overweight |
|  |  |  | Diabetes | Diabetes | Diabetes |
|  |  |  | Cancer | Cancer | Cancer |
|  |  |  | Accidents with injuries | Accidents with injuries | Accidents with injuries |
|  |  |  | Depression | Depression | Depression |
|  |  |  | Anxiety | Anxiety | Anxiety |
|  |  |  | Mental disorders, like schizophrenia | Mental disorders, like schizophrenia | Other mental or psychiatric disorders |
|  |  |  | Epilepsy | Epilepsy | Epilepsy |
|  |  |  | Self-harm or injury | Self-harm or injury | Self-harm or injury |
|  |  |  | Suicide attempt | Suicide attempt | Suicide attempt |
|  |  |  | I was admitted at an advanced stage of a health problem because the health problem had not been recognized at an earlier stage | The adult was admitted at an advanced stage of a health problem because the health problem had not been recognized at an earlier stage | Person is admitted at an advanced stage of a health problem because the health problem had not been recognized at an earlier stage |
|  |  |  | I was ill and needed medical care for a previously diagnosed condition | The adult became ill and needed medical care for a previously diagnosed condition because adult can’t follow the treatment very well | Person becomes ill and needs medical care for a previously diagnosed condition because adherence to treatment is poor |
| N/A | Thinking of the reasons for hospital contacts in autistic adults, select 3 options below that you think might be top considerations for research to promote better health and prevent hospital contacts in adults with autism. (Please tick 3 boxes). | Select 3 options below that you think might be top considerations for research to promote better health and prevent hospital contacts in adults on the autism spectrum. (Please, tick 3 boxes). | I don’t think that I can make a choice | N/A | I prefer to not make a choice |
|  |  |  | Research into regular wellness checkups in adults with autism spectrum |  | Research on the frequency of regular wellness checkups in adults with autism spectrum |
|  |  |  | Research on ways to improve the primary care provider’s awareness of health risks in adults with autism spectrum |  | Research on ways to improve the primary care provider's awareness of health risks in adults with autism spectrum |
|  |  |  | Research on education programs for adults with autism, their carers and partners to raise their awareness of health risks |  | Research on education programs for adults with autism, their carers and partners to raise their awareness of health risks |
|  |  |  | Research on early warning signs of illness or poor health in adults with autism spectrum |  | Research on early warning signs of illness or poor health in adults with autism spectrum |
|  |  |  | Research on health promotion approaches for adults with autism spectrum |  | Research on health promotion approaches for adults with autism spectrum |
|  |  |  | Research into ways to help adults with autism follow health care recommendations |  | Research on how well adults with autism follow health care recommendations |
|  |  |  | Research on making a person's 'health profile' part of an 'autism passport' that adults with autism spectrum can carry with them |  | Research on making a person's 'health profile' part of an 'autism passport' that adults with autism spectrum can carry with them |
|  |  |  | Research on adjustments that make health care services more user-friendly for adults with autism spectrum |  | Research on adjustments that make health care services more user-friendly for adults with autism spectrum |
|  |  |  | Other, please fill in |  | Other, please fill in |
| Do you know of an organization or clinician, in your area or elsewhere in your country, which has a way to monitor health and prevent poor health conditions that works well for adults with autism spectrum? | Do you know of an organization or professional, in your area or elsewhere in your country, which has a way to monitor health and prevent poor health conditions that works well for adults with autism spectrum? | Do you know of an organization or clinician, in your area or elsewhere in your country, which has developed a way to monitor health and prevent poor health conditions that works well for adults on the autism spectrum? | Yes | Yes | Yes |
|  |  |  | No | No | No |
|  |  |  | Don’t know | Don’t know | Don’t know |
| If yes, what is its name? Fill in blank (if you know more than 1, name your first choice) | If yes, what is its name? Fill in blank (if you know more than 1, name your first choice) | If yes, what is its name? Fill in blank (if you know more than 1, name your first choice) | _____ | _____ | _____ |
| If yes, what is its location? Fill in blank (if you know more than 1, name your first choice) | If yes, what is its location? Fill in blank (if you know more than 1, name your first choice) | If yes, what is its location? Fill in blank (if you know more than 1, name your first choice) | _____ | _____ | _____ |

*Note*. N/A = Question not available for the correspondent group.

**Table S2**

*Demographic background information of the respondents: adults, carers and professionals*

| Characteristic | Answer | Autistic adult  (N=522) |  | Carer / Cared-for adult  (N=442) |  | Professional  (N=113) |
| --- | --- | --- | --- | --- | --- | --- |
| Gender | Female | 346 (66.3) |  | 362 (81.9) / 125 (28.3) |  | 77 (68.1) |
|  | Male | 157 (30.1) |  | 79 (17.9) / 316 (71.5) |  | 34 (30.1) |
|  | Other or no answer | 19 (3.6) |  | 1 (0.2) / 1 (0.2) |  | 2 (1.8) |
| Age (years) | 18-25 | 118 (22.6) |  | 7 (1.6) / 247 (55.9) |  | N/A |
|  | 26-35 | 176 (33.7) |  | 20 (4.5) / 118 (26.7) |  |  |
|  | 36-45 | 116 (22.2) |  | 46 (10.4) / 51 (11.5) |  |  |
|  | 46-55 | 88 (16.9) |  | 187 (42.3) / 21 (4.8) |  |  |
|  | 56-64 | 21 (4.0) |  | 117 (26.5) / 4 (0.9) |  |  |
|  | > 65 | 3 (0.6) |  | 65 (14.7) / 1 (0.2) |  |  |
| Living area | | | | | | |
| Country | Denmark | 214 (41.0) |  | 134 (30.3) |  | 20 (17.7) |
|  | France | 69 (13.2) |  | 68 (15.4) |  | 26 (23.0) |
|  | Finland | 66 (12.6) |  | 36 (8.1) |  | 25 (22.1) |
|  | Spain | 25 (4.8) |  | 83 (18.8) |  | 12 (10.6) |
|  | Poland | 44 (8.4) |  | 41 (9.3) |  | 3 (2.7) |
|  | Italy | 42 (8.1) |  | 30 (6.8) |  | 14 (12.4) |
|  | Iceland | 19 (3.6) |  | 42 (9.5) |  | 6 (5.3) |
|  | United Kingdom | 23 (4.4) |  | 0 |  | 6 (5.3) |
|  | Republic of Ireland | 14 (2.7) |  | 8 (1.8) |  | 0 |
|  | German | 6 (1.2) |  | 0 |  | 0 |
|  | Portugal | 0 |  | 0 |  | 1 (0.9) |
| Living area | Capital city | 127 (24.3) |  | 155 (35.1) |  | 42 (37.2) |
|  | Other than a capital city | 395 (75.7) |  | 287 (64.9) |  | 71 (62.8) |
| Community size | < 1.000 people | 33 (6.3) |  | 32 (7.2) |  | 4 (3.5) |
|  | 1.000-20.000 people | 103 (19.7) |  | 97 (21.9) |  | 12 (10.6) |
|  | 20.000-100.000 people | 150 (28.7) |  | 117 (26.5) |  | 32 (28.3) |
|  | 100.000-1.000.000 | 140 (26.8) |  | 116 (26.2) |  | 46 (40.7) |
|  | > 1.000.000 | 50 (9.6) |  | 58 (13.1) |  | 19 (16.8) |
|  | Don't Know | 46 (8.8) |  | 22 (5.0) |  | 0 |
| Education | | | | | | |
| Adult is attending school, home school or education program now | Yes, full time | 67 (12.8) |  | N/A |  | N/A |
|  | Yes, part time | 53 (10.2) |  |  |  |  |
|  | No | 402 (77.0) |  |  |  |  |
| If yes, actual education level | Primary level school | 3 (2.5) |  | N/A |  | N/A |
|  | Secondary level school | 29 (24.2) |  |  |  |  |
|  | Technical, vocational or job training school | 18 (15.0) |  |  |  |  |
|  | College or University | 70 (58.3) |  |  |  |  |
| If no, completed education level | Primary level school | 44 (11.0) |  | N/A |  | N/A |
|  | Secondary level school | 73 (18.2) |  |  |  |  |
|  | Technical, vocational or job training school | 99 (24.6) |  |  |  |  |
|  | College or University | 166 (41.3) |  |  |  |  |
|  | Don't Know | 20 (5.0) |  |  |  |  |
| Adult remember the age when completing education | Yes | 369 (91.8) |  | N/A |  | N/A |
|  | No | 33 (8.2) |  |  |  |  |
| Adult age at completed education | Mean + Standard deviation [range] | 24,3 ± 7.8[3-52] |  | N/A |  | N/A |
| Carer years of education | < 10 years | N/A |  | 47 (10.6) |  |  |
|  | 10-12 years |  |  | 32 (7.2) |  |  |
|  | 13-16 years |  |  | 102 (23.1) |  |  |
|  | >16 years |  |  | 224 (50.7) |  |  |
|  | Other |  |  | 37 (8.4) |  |  |
| Employment | | | | | | |
| Current employment status | Unemployed | 271 (51.9) |  | 50 (11.3) |  | N/A |
|  | Employed (part time o full time) | 179 (34.3) |  | 229 (51.8) |  |  |
|  | Self-employed | 26 (5.0) |  | 45 (10.2) |  |  |
|  | Student | N/A |  | 9 (2.0) |  |  |
|  | Volunteer | 46 (8.8) |  | 14 (3.2) |  |  |
|  | Retired | N/A |  | 95 (21.5) |  |  |
| If unemployed, reasons for unemployment | A disability that prevents from having a job | 88 (32.5) |  | N/A |  | N/A |
|  | Student | 44 (16.2) |  |  |  |  |
|  | Looking for a job | 35 (12.9) |  |  |  |  |
|  | Believing that she/he cannot find a job | 22 (8.1) |  |  |  |  |
|  | Retired | 20 (7.4) |  |  |  |  |
|  | Other | 62 (22.9) |  |  |  |  |
| Age at diagnosis | | | | | | |
| Age at autistic diagnosis | 16-25 | 69 (20.5) |  | 40 (58.0) |  | N/A |
|  | 26-35 | 122 (36.3) |  | 15 (21.8) |  |  |
|  | 36-45 | 85 (25.3) |  | 9 (13.0) |  |  |
|  | 46-55 | 50 (14.9) |  | 5 (7.2) |  |  |
|  | >=56 | 10 (3.0) |  | 0 (0) |  |  |
| Carers’ relationship with the adult | | | | | | |
| Years of knowledge of the adult | Less than 1 year | N/A |  | 2 (0.5) |  | N/A |
|  | 1-5 years |  |  | 8 (1.8) |  |  |
|  | 5-10 years |  |  | 8 (1.8) |  |  |
|  | More than 10 years but not the adult's whole life |  |  | 31 (7.0) |  |  |
|  | The adult's whole life |  |  | 393 (88.9) |  |  |
| Relationship with the adult | Parent | N/A |  | 381 (86.2) |  | N/A |
|  | Other family member related by blood |  |  | 29 (6.6) |  |  |
|  | Spouse or partner |  |  | 16 (3.6) |  |  |
|  | A carer, but not a family member, spouse or partner |  |  | 16 (3.6) |  |  |
| Level of independence of the autistic adult | High level of independence | N/A |  | 38 (8.6) |  | N/A |
|  | Some independence but needs support |  |  | 173 (39.1) |  |  |
|  | Needs a high level of support in daily living |  |  | 155 (35.1) |  |  |
|  | Needs high level institution-like care |  |  | 76 (17.2) |  |  |
| Professionals’ backgrounds and characteristics of their workplace | | | | | | |
| Professional Knowledge of or current work experience in health conditions, health behaviors and medical contacts in autistic adults | Yes | N/A |  | N/A |  | 113 (65.7) |
|  | No |  |  |  |  | 59 (34.3) |
| Professional type | Psychologist | N/A |  | N/A |  | 39 (34.5) |
|  | Other |  |  |  |  | 18 (15.9) |
|  | Teacher/pedagogue |  |  |  |  | 12 (10.6) |
|  | Psychiatrist |  |  |  |  | 12 (10.6) |
|  | Nurse |  |  |  |  | 8 (7.1) |
|  | General practitioner |  |  |  |  | 7 (6.2) |
|  | Social worker |  |  |  |  | 6 (5.3) |
|  | Physical or occupational therapist |  |  |  |  | 2 (1.8) |
|  | Teaching assistant/nursery assistant |  |  |  |  | 3 (2.7) |
|  | Medical specialist, other than psychiatrist |  |  |  |  | 4 (3.5) |
|  | Other medical professional (open answer) |  |  |  |  | 1 (0.9) |
|  | Mental health therapist |  |  |  |  | 1 (0.9) |
|  | Criminal justice (e.g. police, courts, legal advocate) |  |  |  |  | 0 |
| Years in jobs in adult services and care | <1 year | N/A |  | N/A |  | 2 (1.8) |
|  | 1-2 years |  |  |  |  | 9 (8.0) |
|  | 3-5 years |  |  |  |  | 12 (10.6) |
|  | 6-10 years |  |  |  |  | 30 (26.6) |
|  | >10 years |  |  |  |  | 60 (53.1) |
| Source of experience and knowledge about services for adults | Current job location (e.g., capital city or small town) | N/A |  | N/A |  | 39 (34.5) |
|  | Current job location and a wider area (e.g., the region or state where your job is located) |  |  |  |  | 54 (47.8) |
|  | Most closely connected to the whole country |  |  |  |  | 20 (17.7) |

*Note*. N/A = Question not available for the correspondent group. Values expressed as number of responders and frequencies (in parenthesis).

**Table S3**

*Gender differences in self-reported affirmative answers on health profile of autistic adults and carers’ autistic adults*

| Answer | Autistic adult | | | |  | Carer’s autistic adult | | | |
| --- | --- | --- | --- | --- | --- | --- | --- | --- | --- |
|  | Female, n (%) | Male, n (%) | Total, n (%) | Chi square test of independence, p-value |  | Female, n (%) | Male, n (%) | Total, n (%) | Chi square test of independence |
| Sleep problems | 195 (58.4) | 65 (43.3) | 260 (53.72) | χ^2^ = 9.43, *p* = .002 |  | 67 (54.47) | 152 (49.35) | 219 (50.81) | χ^2^ = .92, *p* = .337 |
| GI problem | 167 (49.6) | 54 (35.8) | 221 (45.29) | χ^2^ = 8.01, *p* = .005 |  | 51 (42.50) | 107 (36.15) | 158 (37.98) | χ^2^ = 1.46, *p* = .227 |
| Infection | 154 (46.4) | 36 (23.7) | 190 (39.26) | χ^2^ = 22.53, *p* < .0001 |  | 41 (34.45) | 78 (25.41) | 119 (27.93) | χ^2^ = 3.49, *p* = .062 |
| Allergy | 142 (42.1) | 44 (28.6) | 186 (37.88) | χ^2^ = 8.27, *p* = .004 |  | 45 (36.89) | 91 (29.84) | 136 (31.85) | χ^2^ = 1.99, *p* = .158 |
| Asthma | 55 (16.3) | 17 (11.0) | 72 (14.63) | χ^2^ = 2.44, *p* = .119 |  | 11 (9.02) | 34 (11.00) | 45 (10.44) | χ^2^ = .37, *p* = .543 |
| COPD | 5 (1.5) | 6 (3.9) | 11 (2.23) | χ^2^ = 2.85, *p* = .092 |  | 1 (0.82) | 1 (0.32) | 2 (0.47) | χ^2^ = .46, *p* = .496 |
| Cancer | 11 (3.2) | 3 (2.0) | 14 (2.82) | χ^2^ = 0.62, *p* = .430 |  | 0 (0.00) | 3 (0.97) | 3 (0.70) | χ^2^ = 1.21, *p* = .272 |
| Hypertension | 47 (13.9) | 29 (19.3) | 76 (15.54) | χ^2^ = 2.37, *p* = .124 |  | 5 (4.07) | 16 (5.39) | 21 (5.00) | χ^2^ = .32, *p* = .572 |
| Overweight | 123 (36.3) | 43 (28.3) | 166 (33.81) | χ^2^ = 3.00, *p* = .083 |  | 43 (34.68) | 72 (23.38) | 115 (26.62) | χ^2^ = 5.77, *p* = .016 |
| Diabetes | 10 (2.9) | 10 (6.5) | 20 (4.04) | χ^2^ = 3.56, *p* = .059 |  | 3 (2.44) | 12 (3.90) | 15 (3.48) | χ^2^ = .56, *p* = .456 |
| Depression | 255 (75.9) | 77 (51.3) | 332 (68.31) | χ^2^ = 28.90, *p* < .0001 |  | 53 (44.17) | 101 (34.12) | 154 (37.02) | χ^2^ = 3.70, *p* = .055 |
| Anxiety | 240 (72.5) | 82 (55.8) | 322 (67.65) | χ^2^ = 13.68, *p* < .0001 |  | 74 (61.16) | 176 (58.47) | 250 (59.24) | χ^2^ = .26, *p* = .612 |
| Other mental or psychiatric conditions | 61 (18.5) | 15 (10.3) | 76 (15.97) | χ^2^ = 5.09, *p* = .024 |  | 20 (17.39) | 23 (7.59) | 43 (10.29) | χ^2^ = 8.68, *p* = .003 |
| Epilepsy | 14 (4.1) | 12 (7.9) | 26 (5.26) | χ^2^ = 3.05, *p* = .081 |  | 16 (13.11) | 50 (16.34) | 66 (15.42) | χ^2^ = .70, *p* = .404 |
| Learning disability | 55 (16.8) | 40 (27.6) | 95 (20.08) | χ^2^ = 7.33, p = .007 |  | 48 (39.67) | 100 (33.22) | 148 (35.07) | χ^2^ = 1.58, *p* = .209 |
| ADHD | 91 (27.3) | 29 (19.7) | 120 (24.95) | χ^2^ = 3.08, *p* = .079 |  | 28 (23.73) | 62 (24.45) | 90 (22.11) | χ^2^ = .25, *p* = .616 |
| Accident | N/A | | | |  | 20 (16.26) | 70 (22.58) | 90 (20.79) | χ^2^ = 2.14, *p* = .144 |
| Self-harm/injury | N/A | | | |  | 47 (39.17) | 98 (32.24) | 145 (34.20) | χ^2^ = 1.84, *p* = .175 |
| Suicide attempt | N/A | | | |  | 8 (6.56) | 12 (3.93) | 20 (4.68) | χ^2^ = 1.34, *p* = .247 |

*Note*. GI = Gastrointestinal; COPD = Chronic obstructive pulmonary disease; ADHD = Attention Deficit Hyperactivity Disorder. Values expressed as number of responders and frequencies (in parenthesis). For autistic adults, the question was the following: ‘*Since you became an adult (that is, after your 18 years birthday) have you been diagnosed by a doctor with:’.* For carers the question was the following……

**Table S4**

*Differences between medical and non-medical professionals in affirmative answers on health profile of autistic adults*

| Answer | Medical | Non-medical | Total, n (%) | Chi square test of independence, p-value |
| --- | --- | --- | --- | --- |
| Sleep problems | 21 (84.0) | 80 (90.9) | 101 (89.4) | χ^2^ = 4.51, *p* = .105 |
| GI problem | 18 (72.0) | 51 (58.0) | 69 (61.1) | χ^2^ = 3.51, *p* = .173 |
| Infection | 7 (28.0) | 9 (10.2) | 16 (14.2) | χ^2^ = 7.14, *p* =.028 |
| Allergy | 7 (28.0) | 15 (17.1) | 22 (19.5) | χ^2^ = 8.27, *p* = .004 |
| Asthma | 2 (8.0) | 8 (9.1) | 10 (8.9) | χ^2^ = 3.70, *p* = .158 |
| COPD | 0 | 1 (1.1) | 1 (0.9) | χ^2^ = 2.57, *p* = .276 |
| Cancer | 1 (4.0) | 1 (1.1) | 2 (1.8) | χ^2^ = 3.35, *p* = .187 |
| Hypertension | 4 (16.0) | 9 (10.2) | 13 (11.5) | χ^2^ = 12.48, *p* = .002 |
| Overweight | 12 (48.0) | 30 (34.1) | 42 (37.2) | χ^2^ = 1.92, *p* = .383 |
| Diabetes | 7 (28.0) | 9 (10.2) | 16 (14.2) | χ^2^ = 6.83, *p* = .033 |
| Depression | 21 (84.0) | 68 (77.3) | 89 (78.8) | χ^2^ = 0.64, *p* =.727 |
| Anxiety | 25 (100.0) | 84 (95.5) | 109 (96.5) | χ^2^ = 1.18, *p* =.555 |
| Other mental or psychiatric conditions | 17 (68.0) | 40 (45.5) | 57 (50.4) | χ^2^ = 5.73, *p* = .057 |
| Epilepsy | 21 (84.0) | 62 (70.5) | 83 (73.5) | χ^2^ = 4.25, *p* = .119 |
| Learning disability | 25 (100.0) | 72 (81.8) | 97 (85.8) | χ^2^ = 5.30, p = .071 |
| ADHD | 19 (76.0) | 62 (70.5) | 81 (71.7) | χ^2^ = 2.69, *p* = .261 |
| Suicide or suicide attempt | 11 (44.0) | 32 (36.4) | 43 (38.1) | χ^2^ = 1.56, *p* = .459 |
| Self-harm or injury | 19 (76.0) | 78 (88.6) | 97 (85.8) | χ^2^ = 5.34, *p* = .069 |
| Accidents with injuries | 16 (64.0) | 43 (48.9) | 59 (52.2) | χ^2^ = 2.39, *p* = .303 |

*Note*. GI = Gastrointestinal; COPD = Chronic obstructive pulmonary disease; ADHD = Attention Deficit Hyperactivity Disorder. Values expressed as number of responders and frequencies (in parenthesis). The question was the following: ‘*Based on your professional knowledge or work experience, do you believe that the following conditions are more frequent in autistic adults, compared to adults not on the autism spectrum?’.*
